# Neuraminidase of influenza A viruses induces global desialylation of host cells via its intracellular function

**DOI:** 10.1128/spectrum.03328-25

**Published:** 2026-02-18

**Authors:** Daiki Kobayashi, Takahiro Hiono, Norikazu Isoda, Yoshihiro Sakoda

**Affiliations:** 1 Laboratory of Microbiology, Department of Disease Control, Faculty of Veterinary Medicine, Hokkaido University 12810, Sapporo, Hokkaido, Japan; 2 One Health Research Center, Hokkaido University 12810, Sapporo, Hokkaido, Japan; 3 Hokkaido University Institute for Vaccine Research and Development (HU-IVReD), Hokkaido University 12810, Sapporo, Hokkaido, Japan; 4 International Collaboration Unit, International Institute for Zoonosis Control, Hokkaido University 12810, Sapporo, Hokkaido, Japan; Universiteit Utrecht, Utrecht, the Netherlands

**Keywords:** influenza A virus, neuraminidase, glycan recapping, sialic acid, glycobiology

## Abstract

The neuraminidase protein (NA) of influenza A viruses (IAVs) plays a role in the release of viruses from infected cells. NA on viral particles hydrolyzes sialylated glycans on the cell surface for viral budding. However, the maturation and intracellular functions of NA are poorly understood. To investigate how NA functions intracellularly, glycans displayed on IAV-infected cells were profiled by lectins, and global glycome alterations, accompanied by exposed terminal galactose and glycan recapping with α1-2 fucose, were found in the virus-infected cells. Since α1-2 fucosyltransferases are localized in the Golgi, these unique structures suggest a potential NA function in the IAV-infected cells. Functional analyses using antivirals and NA-expressing cells indicate that intracellular NA function is necessary for the glycome alterations. Time-course analyses in IAV-infected cells revealed that global desialylation and α1-2 fucosylation could be observed 5 h post-inoculation, corresponding to the timeframe of viral protein expression. These observations provide a novel theory of NA functions that NA obtains its enzymatic activity intracellularly before virus assembly and serves desialylated glycans for competitive glycosyltransferases, including α1-2 fucosyltransferases, as their acceptors, resulting in glycan recapping with α1-2 fucose. Hence, intracellular NA blocks the re-sialylation of glycans, promoting efficient virus release from infected cells by inhibiting the interaction between progeny virions and sialosides. This study further demonstrated the potential NA functions of limiting secondary IAV infection. These findings provide insights into the evolutionary strategies of IAVs for shaping the strict window of superinfection by NA functions under a balance between successful replication and reassortment.

**IMPORTANCE:**

Influenza A viruses (IAVs) exploit glycans for their replication cycle. The hemagglutinin protein uses sialic acid for viral attachment, and the neuraminidase protein (NA) hydrolyzes sialosides for virus release. However, the intracellular functions of NA are not well understood. This study demonstrated that intracellular NA induces global desialylation and glycan recapping with unique structures in IAV-infected cells. This suggests a novel mode of NA function during the IAV lifecycle, where virus particles are ready to be released at the assembly, and NAs no longer need to hydrolyze the sialic acids upon egress from the cells. Therefore, the present study provides novel and significant insights into the fundamental understanding of the lifecycle of IAV. Furthermore, as NA is a primary target for anti-influenza drugs, understanding the mechanism of intracellular NA function may also support the development of antivirals.

## INTRODUCTION

Influenza A viruses (IAVs), which belong to the family *Orthomyxoviridae* and genus *Alphainfluenzavirus*, contain the viral proteins hemagglutinin (HA) and neuraminidase (NA) (1). These proteins are essential for viral attachment, entry, and budding. The HA is a viral lectin and facilitates attachment of virions to host cells via specific binding to sialylated glycoconjugates (2). In contrast, NA is a viral glycosidase that recognizes sialic acid at the non-reducing terminus of glycans and hydrolyzes sialosides (3, 4). The main function of NA is to destroy viral receptors upon IAV budding, facilitating efficient viral release from infected cells and spreading the infection to other cells (5). The NA also promotes viral movement on target cells by reducing the density of sialylated glycans and removing “decoy” receptors (6–9). As both HA and NA recognize the same sialylated glycans during viral replication, destruction of receptor molecules by NA potentially limits superinfection and reassortment of IAVs at the cellular level (10, 11). As a virus-specific enzyme, NA is an attractive target for the development of anti-influenza drugs such as oseltamivir and zanamivir (12, 13). These drugs aggregate virus particles at the cellular surface of infected cells (14), leading to the indispensability of viral NA function during the IAV lifecycle, especially upon virus budding.

IAV envelope and host proteins are glycosylated by *N*-glycans. During *N*-glycan synthesis, *en bloc* transfer of an *N*-glycan progenitor to N-!P-S/T (!P: any amino acid other than proline) sequon motif occurs in the endoplasmic reticulum (ER) membrane. Glycans are sequentially processed in the ER or Golgi lumen by host glycosidases and glycosyltransferases (15). Viral infection sometimes induces atypical glycan modification of the host cells, as well as of viral proteins, by modulating host glycan synthesis machinery. For example, herpes simplex virus type 1 infection upregulates fucosyltransferase (FUT3, FUT5, and FUT6) genes via the NF-κB signaling pathway, leading to high levels of sialyl Lewis X on infected cells (16, 17). In contrast, *N*-glycans in IAV-infected cells and viral glycoproteins are broadly desialylated due to viral NA activity (18). Recent glycobiological approaches using lectins have suggested global glycome alterations in IAV-infected cells (19, 20). Glycans displayed on IAV particles propagated in Madin-Darby canine kidney (MDCK) cell lines and those on IAV-infected cells have been recognized by EEL (specific for α1-3 galactose) or TJA-II (specific for α1-2 fucose), in addition to ECA (specific for terminal LacNAc), suggesting alternative recapping of glycan terminals (19–21). These unique glycan profiles have also been confirmed by a glycomic analysis using liquid chromatography-mass spectrometry, which indicates α1-3 galactosylation and α1-2 fucosylation at the non-reducing terminus of glycans on HA and NA (22). Because α1-3 galactosyltransferases and α1-2 fucosyltransferases are localized in the Golgi apparatus and function on non-sialylated glycans (23, 24), two hypotheses may support the molecular mechanisms underlying the IAV infection-induced glycosylation alterations: (i) IAV infections disrupt the normal harmony of glycan synthesis machinery in host cells to upregulate α1-3 galactosyltransferase and/or α1-2 fucosyltransferase genes, or (ii) NA intracellularly liberates the terminal sialic acids to generate the terminal LacNAc, as an acceptor of α1-3 galactosyltransferase and/or α1-2 fucosyltransferases (20).

To demonstrate the intracellular function of NA in IAV-infected cells, the present study first focused on the glycan profiles of IAV-infected MDCK cells and demonstrated glycan recapping by α1-2 fucose on IAV-infected cells, which was supported by the glycan profiles of NA-expressing cells. Functional analyses indicated that glycan recapping was induced by the intracellular function of NA in IAV-infected cells, highlighting the novel mechanisms of fundamental NA functions in promoting virus release from cells. Moreover, this study demonstrates the potential contribution of NA functions to superinfection exclusion in IAVs. The findings provide a novel insight into NA functions in the evolutionary strategy of IAVs.

## RESULTS

### Changes in the glycan profiles on the surface of MDCK cells infected with an IAV

**Fig 1:**
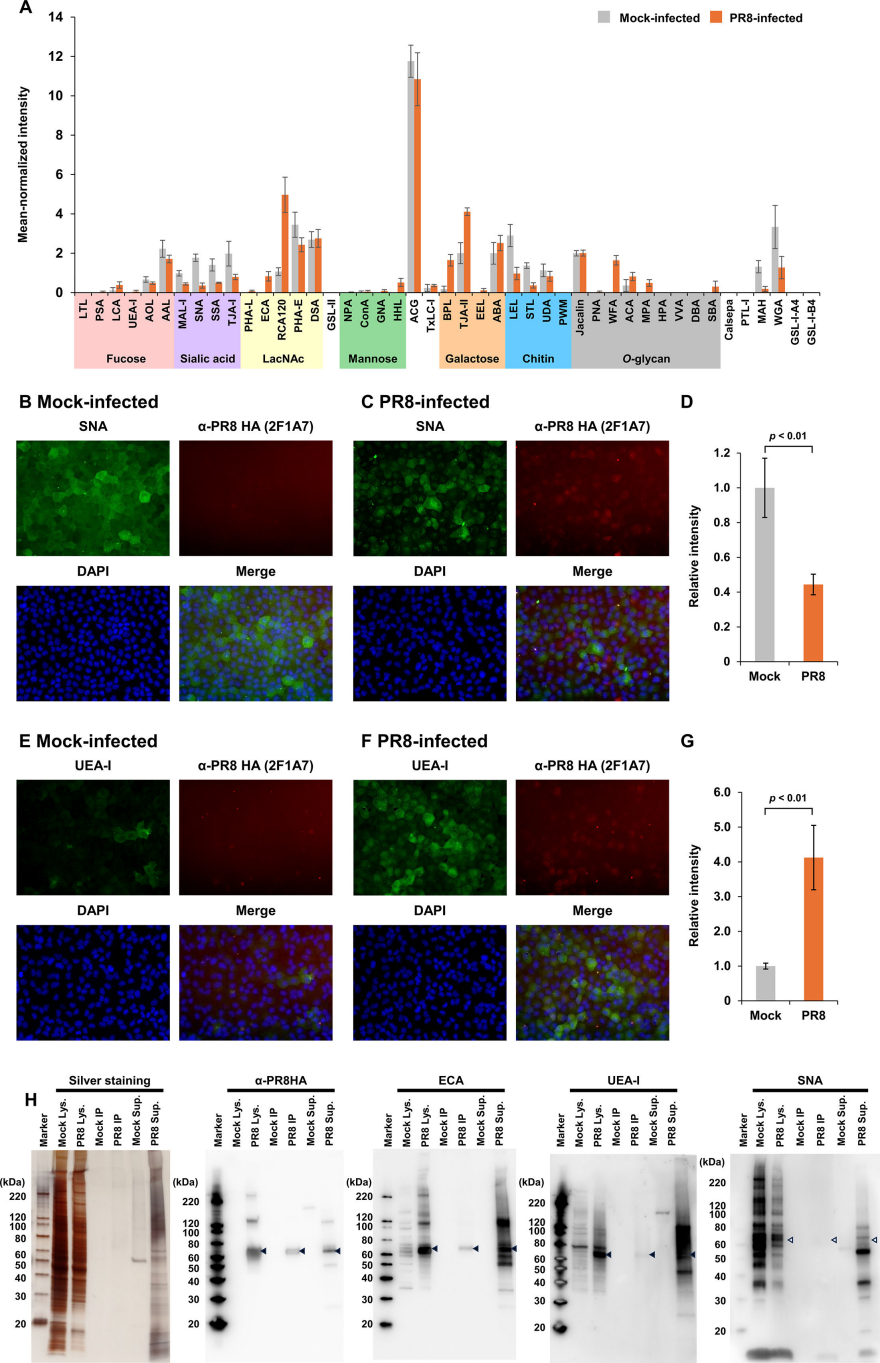
Alterations in glycan profiles of MDCK cells were broadly observed on both host and viral proteins during PR8 infection. (**A**) The surface of MDCK cells infected with mock (gray bars) and PR8 (orange bars) was labeled with biotin, and the whole-cell lysates were placed on lectin microarray slides to incubate with a set of lectins. The interactions of glycans displayed on the cellular surfaces and lectins were detected with a fluorescent-labeled secondary antibody. The data are mean-normalized and represented as mean signals of three spots ± standard deviations. (**B and C**) Lectin and immunofluorescence staining of mock- or PR8-infected MDCK cells at 8 hpi. Cells were stained with SNA (green) and an anti-PR8 HA monoclonal antibody (red) and counterstained with 4′,6-diamidino-2-phenylindole (DAPI) (blue). (**D**) Fluorescent intensities derived from SNA in an average of three fields of view were compared between mock- and PR8-infected cells. The signal intensity of PR8-infected cells was significantly lower than that of mock-infected ones (*P* < 0.01, Student’s *t*-test). (**E and F**) Cells were stained with UEA-I (green) and an anti-PR8 HA monoclonal antibody (red) and counterstained with DAPI (blue). (**G**) Fluorescent intensities derived from UEA-I in an average of three fields of view were compared between mock- and PR8-infected cells. The signal intensity of PR8-infected cells was significantly higher than that of mock-infected ones (*P* < 0.01, Student’s *t*-test). (**H**) Whole-cell lysates (Lys.) and supernatant (Sup.) of mock- and PR8-infected MDCK cells were collected for western blotting and lectin blotting. Whole-cell lysates were immunoprecipitated with the anti-PR8 HA antibody (IP). Those samples were analyzed by SDS-PAGE with silver staining, western blotting with the anti-PR8 HA antibody, and lectin blotting with ECA, UEA-I, and SNA. The filled arrowheads indicate bands at approximately 65 kDa, which are considered HA proteins of PR8. The open arrowheads indicate the expected size of 65 kDa, while no clear bands were observed.

To validate previously observed glycome alterations in MDCK cells infected with IAV (19, 20, 22), glycan structures on the surface of MDCK cells infected with IAV, A/Puerto Rico/8/1934 (H1N1) (PR8), were profiled using a lectin microarray. Briefly, the cellular surface of MDCK cells inoculated with PR8 or mock-infected cells was labeled with hydrophilic biotin-NHS analogs, and the cell lysates were subjected to lectin microarray analysis to assess the reactivity of a series of lectins (20, 25). Cell surface glycan profiles were enriched by detecting biotin-labeled proteins using fluorescently labeled streptavidin (Fig. 1A). Lectins specific to sialylated glycans (MAL-I, SNA, SSA, and TJA-I) exhibited low signal intensities in PR8-infected MDCK cells. In contrast, ECA, RCA120, BPL, and WFA, which preferentially recognize the terminal LacNAc or its cluster (26), exhibited higher signal intensities in PR8-infected cells than mock-infected cells. These results indicated desialylation on the surface of PR8-infected MDCK cells. Next, glycans terminating with α1-3 galactose or α1-2 fucose were examined because previous reports noted that these have been observed in IAV-infected MDCK cells but rarely detected in non-infected cells (20, 22). Although α1-3 galactose had been observed from whole-cell lysates of PR8-infected MDCK cells (20), the EEL signal intensity was not high in the PR8-infected MDCK cells compared to the mock-infected ones in this study (Fig. 1A). In contrast, the higher signal intensity of TJA-II, specific for α1-2 fucose, was observed in PR8-infected cells, consistent with previous studies (20, 22). These results indicate that IAV-infected cells display glycans terminating with α1-2 fucose instead of sialic acids, suggesting a unique glycan profile on IAV-infected cells.

### Global desialylation and α1-2 fucosylation on the surface of IAV-infected MDCK cells

For further validation of whether glycome alteration was specifically observed in virus-infected cells, fluorescent staining was performed with lectins specific for terminal sialic acids (SNA) and α1-2 fucose (UEA-I), in parallel with immunostaining using an anti-PR8 HA antibody. The surface of HA-positive cells was not stained by SNA (Fig. 1C and D), while the mock-infected HA-negative cells were strongly stained by SNA (Fig. 1B and D), indicating desialylation on the surface of HA-positive cells. Conversely, UEA-I signal intensity was higher on the surface of PR8-infected cells than mock-infected cells (Fig. 1E through G). These results demonstrated desialylation and glycan recapping with α1-2 fucose on the HA-positive MDCK cells after PR8 infection.

To investigate whether the changes in glycan profiles in MDCK cells during PR8 infection are commonly observed by other IAV infections, the reactivities of SNA and UEA-I were tested in MDCK cells infected with duck-origin IAVs with NA subtypes from N1 to N9. Eight strains (N1–6, N8, and N9 subtypes) are non-pathogenic avian influenza viruses isolated from ducks, and an N7 strain is a recombinant virus, Vac2-FLAG. Fluorescent intensities of SNA in avian influenza virus-infected cells varied among the strains. Especially, the signals were drastically diminished in cells infected with N1, N4, and N6–8 viruses (Fig. S1). In contrast, a marked increase in UEA-I signal was observed in all cells infected with avian influenza viruses except an N3 virus (Fig. S2). It was noted that the growth of the N3 virus, A/duck/Hokkaido/84/2002 (H5N3), was low in MDCK cells, possibly causing a slight increase in UEA-I signal in the infected cells without a significant difference. These results suggest that the changes in glycan profiles with increased α1-2 fucose can be commonly observed in IAV-infected MDCK cells.

Because IAVs lead to a global decrease in sialic acid and an increase in α1-2 fucose on the surface of infected MDCK cells, a similar assay was attempted using A549 and Vero E6 cell lines. Since PR8 protein expression was not high in those cells, lectin fluorescent staining showed an obvious decrease in SNA signal intensity for both PR8 and Vac2 infections (Fig. S3A through C and G through I). In contrast, an increase in UEA-I signals was not observed (Fig. S3D through F and J through L). Thus, hydrolysis of sialic acid by NA was induced after IAV infection in various types of cells, whereas glycan recapping appears to be highly dependent on the cell-specific glycan synthesis machinery.

The next question was whether the changes in glycan profiles were specific to either viral or host proteins. To assess this, culture supernatants and whole-cell lysates of mock- or PR8-infected cells were subjected to lectin and western blot analyses (Fig. 1H). The total protein concentration in whole-cell lysates was approximately adjusted using the band intensities of silver staining. Viral HA was enriched via immunoprecipitation using an anti-HA antibody. A band of approximately 65 kDa (arrowhead) detected by the anti-HA antibody in the immunoprecipitated sample of the PR8-infected cells was observed by lectin blotting with both lectins specific for LacNAc (ECA) and α1-2 fucose (UEA-I). In contrast, HA protein was not detected in the immunoprecipitated sample with a lectin specific for terminal sialic acids (SNA). This demonstrated that desialylation and glycan recapping with α1-2 fucose are observed on the viral HA. Moreover, lectin blotting of whole-cell lysates and culture supernatants of PR8-infected cells exhibited several higher-intensity bands than those of mock-infected cells using ECA and UEA-I, in addition to a high-intensity band of HA, while the band intensity of SNA in whole-cell lysates was lower in PR8-infected cells. It should be noted that several bands were observed in the culture supernatant of PR8-infected cells when blotting with SNA. This reflects a higher degree of protein contamination in the supernatant of virus-infected cells compared to that of mock-infected cells. These results suggested that some host proteins, as well as viral HA, were globally desialylated and α1-2 fucosylated during PR8 infection in MDCK cells.

### α1-2 fucosylation in IAV-infected cells is induced by the latter part of a viral replication cycle

**Fig 2:**
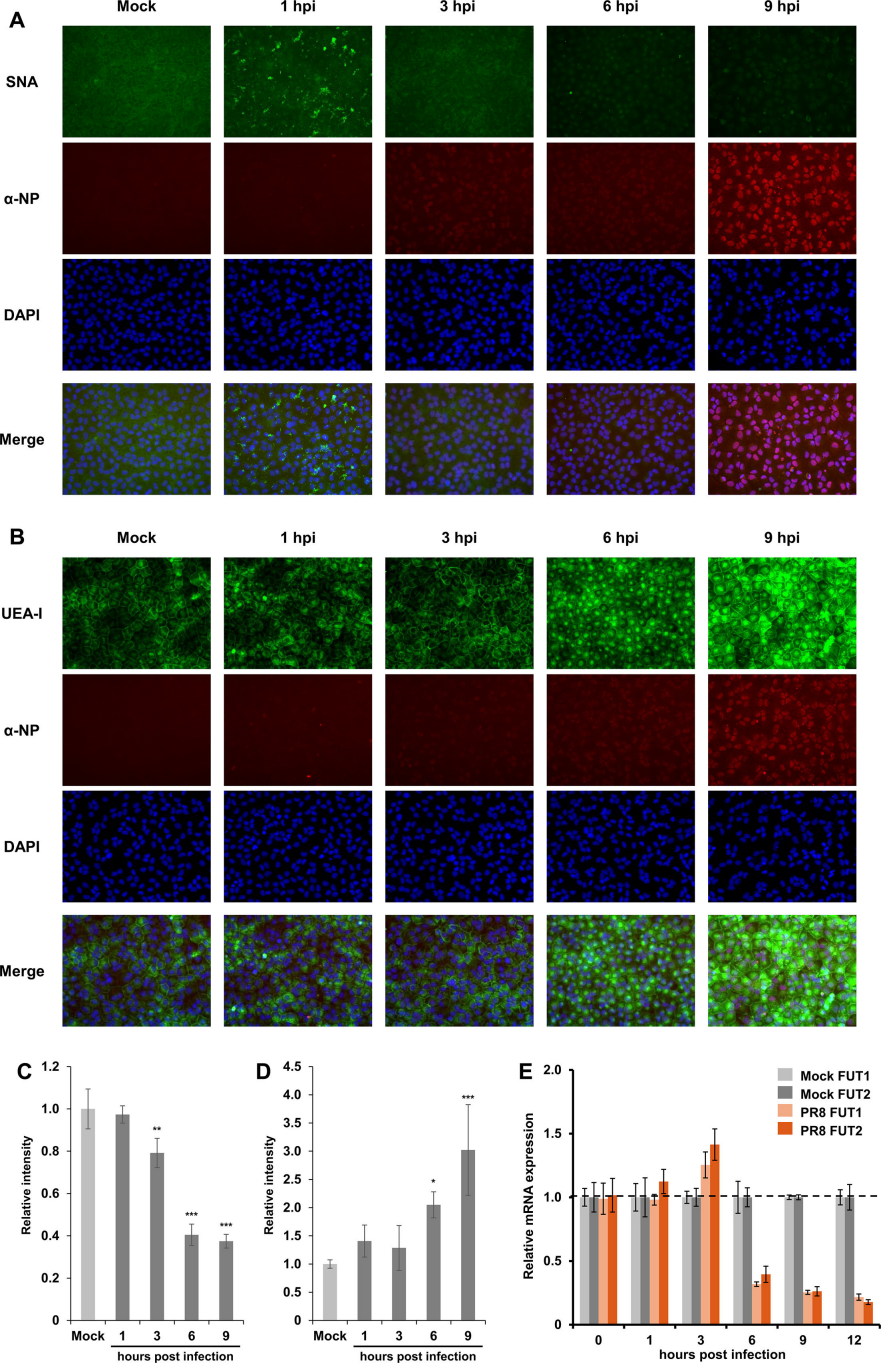
Glycome alterations associated with desialylation and α1-2 fucosylation were induced by the latter stage of the PR8 replication cycle. (**A and B**) PR8-infected MDCK cells were fixed with methanol at multiple time points of 1, 3, 6, and 9 hpi and stained with SNA or UEA-I (green) together with immunostaining with an anti-NP monoclonal antibody (red). Cells were also counterstained with 4′,6-diamidino-2-phenylindole (DAPI) (blue). (**C**) Fluorescent intensities derived from SNA in an average of three fields of view were compared between mock- and PR8-infected cells by one-way ANOVA with Dunnett’s *post hoc* test (*, *P* < 0.05; **, *P* < 0.01; and ***, *P* < 0.001). (**D**) Fluorescent intensities derived from UEA-I in an average of three fields of view were compared between mock- and PR8-infected cells by one-way ANOVA with Dunnett’s *post hoc* test (*, *P* < 0.05; **, *P* < 0.01; and ***, *P* < 0.001). (**E**) mRNA expression levels of α1-2 fucosyltransferase (FUT1 and FUT2) genes were quantified via RT-qPCR. The relative mRNA levels of FUT1 and FUT2 genes were normalized based on the expression level of glyceraldehyde-3-phosphate dehydrogenase gene as a housekeeping gene and were compared between mock- and PR8-infected MDCK cells at multiple time points. The data are presented as the mean expression level of biological triplicates ± SD.

Since PR8 infection induced changes in the glycome of virus-infected MDCK cells, the display of sialic acids and α1-2 fucose on the cells was monitored by time-course analysis from the viral infection (Fig. 2A and B). The cells infected with PR8 at a multiplicity of infection (MOI) of 1 showed a decrease in SNA signals at 3 h post-infection (hpi) and significantly decreased at 6 and 9 hpi (Fig. 2A and C). Conversely, the proportion of cells recognized by UEA-I increased at 6 hpi and showed high-intensity signals in the perinuclear space at 6 and 9 hpi (Fig. 2B and D). Corresponding to these signal changes in SNA and UEA-I, immunostaining with an anti-nucleoprotein (NP) antibody indicated that viral NP was localized in the nucleus of the infected cells at 3 hpi, whereas nuclear export of NP was confirmed at 6 hpi in the latter stage of the IAV replication cycle, corresponding to the timeline for viral protein expression. These results suggested that the latter part of the viral replication cycle, including viral protein expression and assembly, induces glycome alterations in IAV-infected MDCK cells. Next, expression levels of endogenous fucosyltransferase (FUT1 and FUT2) genes, which are responsible for α1-2 fucosylation, were quantified via RT-qPCR in a time course after PR8 infection in MDCK cells. The mRNA levels of both FUT1 and FUT2 increased slightly at 3 hpi, with no significant difference; thereafter, the expression levels remained low until 12 hpi, potentially due to the host protein shutoff by PR8 infection (Fig. 2E). Thus, the increased glycan recapping by α1-2 fucose at 6 hpi or later in the IAV-infected cells could not be due to the upregulation of endogenous FUT1 or FUT2 gene expression.

**Fig 3:**
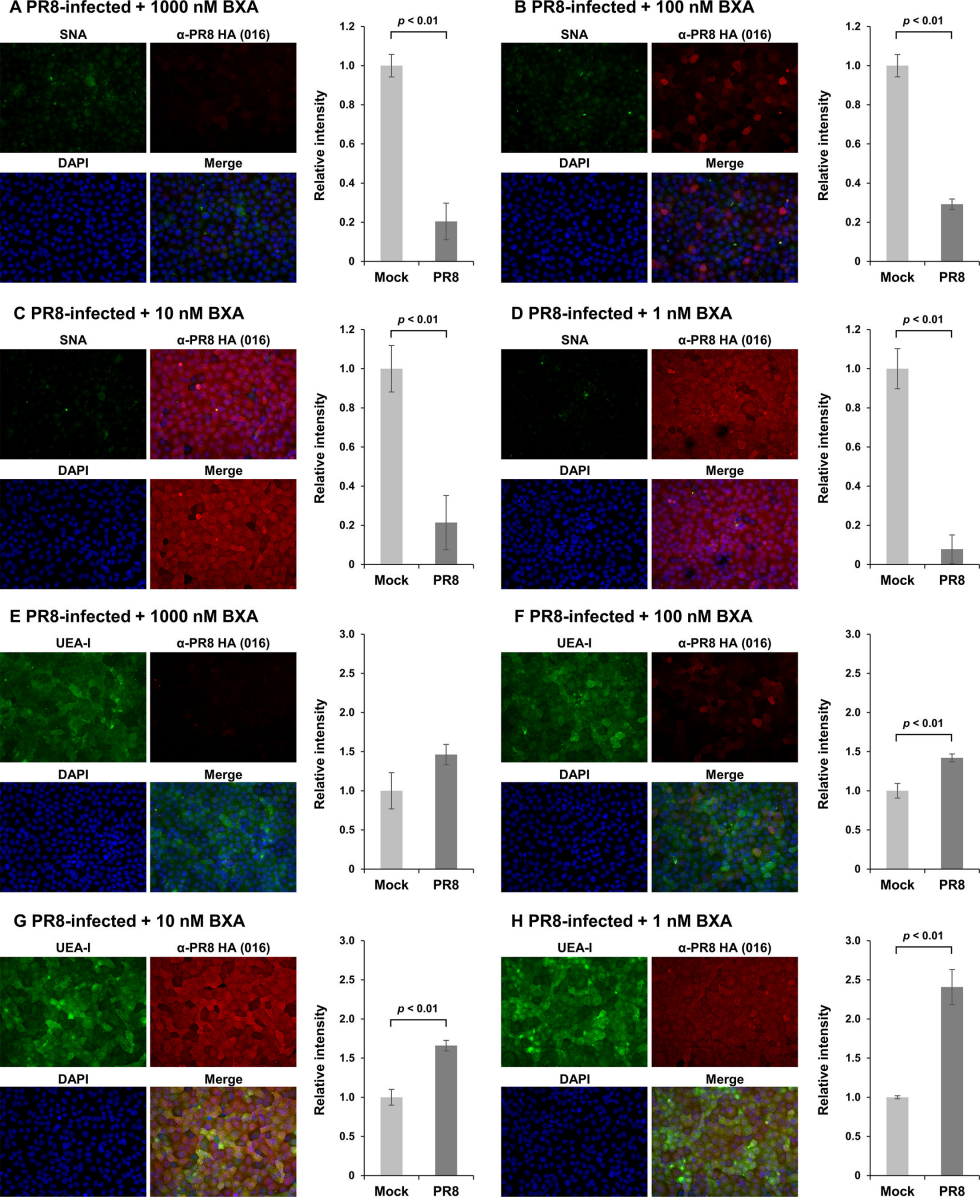
Inhibitory effect of BXA on glycan alterations on PR8-infected MDCK cells. MDCK cells infected with PR8 were cultured in the presence of 1–1,000 nM of BXA for 8 h and subjected to lectin and immunofluorescence staining. The cells were stained with SNA (**A–D**; green) or UEA-I (**E–H**; green) and an anti-PR8 HA monoclonal antibody (red) and counterstained with 4′,6-diamidino-2-phenylindole (DAPI) (blue). Fluorescent intensities derived from SNA or UEA-I in an average of three fields of view were compared between mock- and PR8-infected cells. The statistical analysis was performed with Student’s *t*-test.

The association between glycome alteration in IAV-infected cells and viral protein expression was investigated. Lectin staining was performed on IAV-infected MDCK cells maintained in the presence of a cap-dependent endonuclease inhibitor, baloxavir acid (BXA), to suppress the mRNA synthesis of viral proteins and their subsequent expression (27, 28). All PR8-infected cells showed a decrease in SNA signal intensity, regardless of treatment dose of BXA, compared to the mock-infected cells (Fig. 3A through D). Cells that were faintly stained with an anti-PR8 HA antibody under 1,000 nM of BXA exhibited low UEA-I signal intensity by lectin staining (Fig. 3E). Conversely, HA-positive cells treated with lower concentrations (100, 10, and 1 nM) of BXA exhibited higher UEA-I signal intensities (Fig. 3F through H). These results emphasize that glycome alterations were not caused by IAV entry but at a later stage of the replication cycle, including viral genome transcription, protein expression, or assembly. A similar trend was observed in Vac2-infected MDCK cells, suggesting that these glycome alterations are mediated by a conserved mechanism across IAV strains (Fig. S4). To elucidate the mechanism underlying these glycome changes, the present study focused on the expression and function of NA in IAV-infected cells.

### NA expression in MDCK cells induced desialylation and glycan recapping with α1-2 fucose

**Fig 4:**
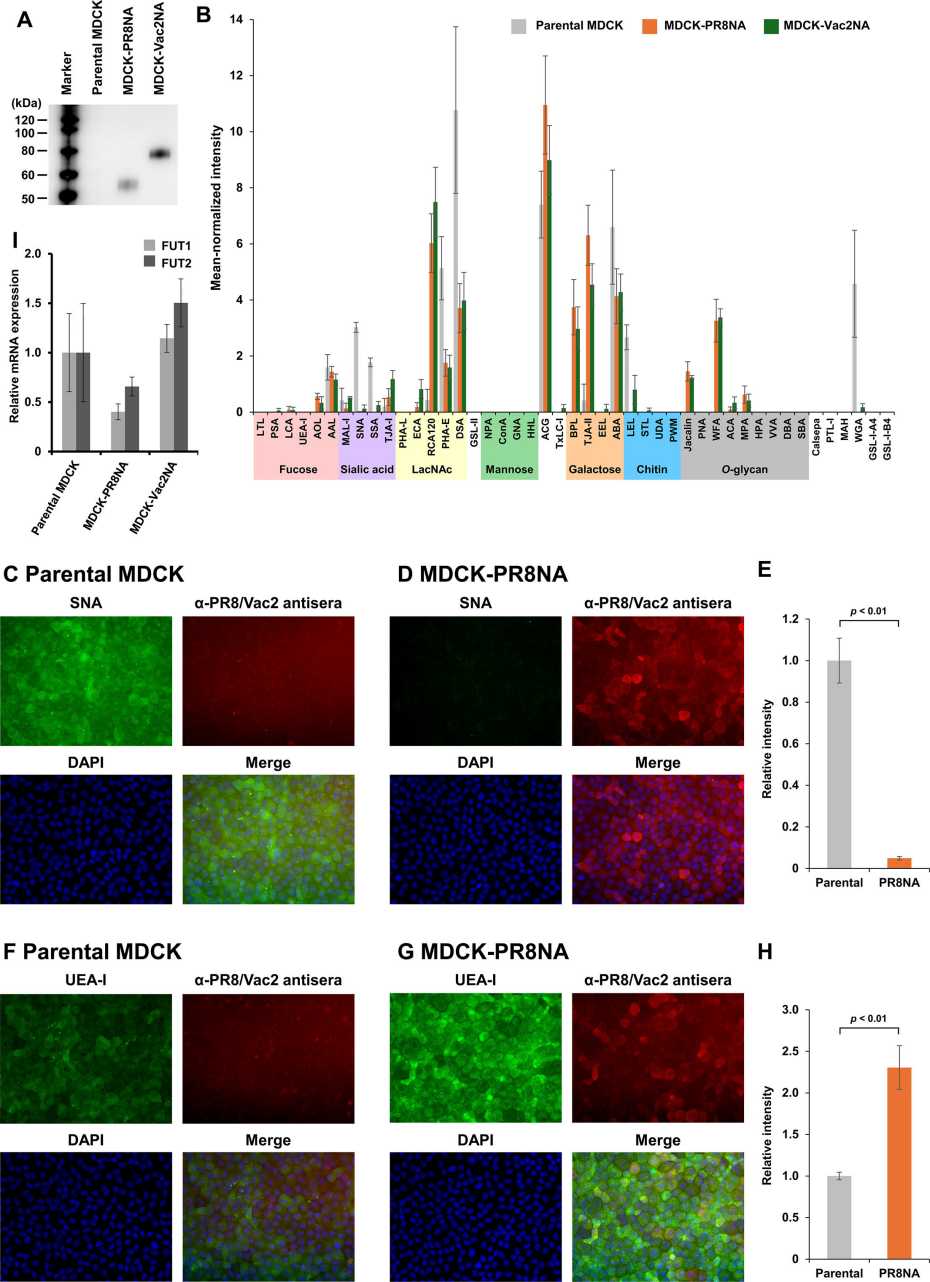
Glycome alterations in MDCK cells stably expressed NA protein of the PR8 or the Vac2 virus. (**A**) Expression of each FLAG-tagged NA protein derived from PR8 or Vac2 was confirmed via SDS-PAGE with western blotting using an anti-DDDDK-tag monoclonal antibody. (**B**) The surface of NA-expressing MDCK cells was labeled with biotin, and whole-cell lysates were placed on lectin microarray slides to incubate with a set of lectins. The interactions of glycans displayed on the cellular surfaces and lectins were detected with a fluorescent-labeled secondary antibody. The data are mean-normalized and presented as mean signals of three spots ± SD. (**C and D**) The wild-type and PR8 NA-expressing MDCK cells were stained with SNA (green) and immunostained with a mixture of PR8 and Vac2 chicken antisera (red) and counterstained with 4′,6-diamidino-2-phenylindole (DAPI) (blue). (**E**) Fluorescent intensities derived from SNA in an average of three fields of view were compared between wild-type and PR8 NA-expressing cells. The statistical analysis was performed with Student’s *t*-test. (**F and G**) The wild-type and PR8 NA-expressing MDCK cells were stained with SNA (green) and immunostained with a mixture of PR8 and Vac2 chicken antisera (red) and counterstained with DAPI (blue). (**H**) Fluorescent intensities derived from SNA in an average of three fields of view were compared between mock- and PR8-infected cells. The statistical analysis was performed with Student’s *t*-test. (**I**) mRNA expression levels of FUT1 and FUT2 genes in the NA-expressing cells were quantified via RT-qPCR. The relative mRNA levels of FUT1 and FUT2 genes were normalized based on the expression level of glyceraldehyde-3-phosphate dehydrogenase gene as a housekeeping gene and were compared between wild-type and PR8 NA-expressing cells. The data are presented as the mean expression level of biological triplicates ± SD.

To investigate the contribution of NA to glycome alterations in IAV-infected cells, MDCK cells stably expressing PR8 NA (MDCK-PR8NA) or Vac2 NA (MDCK-Vac2NA) were established. NA expression in MDCK cells was confirmed by detecting FLAG-fused NA proteins by western blotting with an anti-DDDDK-tag monoclonal antibody (Fig. 4A). A band of either 55 kDa for PR8 NA or 75 kDa for Vac2 NA was detected in each NA-expressing cell via western blotting, indicating the expression of the designed NA in each cloned cell.

The glycan structures on the surface of NA-expressing cells were profiled using a lectin microarray. These NA-expressing cells exhibited glycan profiles similar to those of PR8-infected cells (Fig. 1A and 4B). In particular, lectins specific for sialylated glycans (MAL-I, SNA, and SSA) showed low signal intensities for NA-expressing cells, whereas lectins specific for α1-2 fucose (TJA-II), as well as those specific for terminal LacNAc or its clusters (ECA, RCA120, BPL, WFA), exhibited higher signal intensities for NA-expressing cells than parental MDCK cells. Subsequently, glycome alterations in MDCK-PR8NA or MDCK-Vac2NA cells were also assessed by lectin staining with SNA and UEA-I (Fig. 4C through H; Fig. S5). Both NA-expressing MDCK cells exhibited lower intensity SNA signals and higher intensity UEA-I signals than parental cells. Although the signal intensities of UEA-I were low for both PR8-infected and NA-expressing MDCK cells via lectin microarray, they might have been affected by the orientation or representation of the glycan recognition epitope of this lectin on the microarray slide (Fig. 1A and 4B). Furthermore, the glycan structures recognized by UEA-I were further validated by α1-2 fucosidase pretreatment prior to lectin staining (Fig. S6). To this end, parental MDCK cells, MDCK cells overexpressing α1,3/4 fucosyltransferases (MDCK-FUT cells), and MDCK-PR8NA cells were used (29). The signal intensities of an anti-sialyl Lewis X antibody against MDCK-FUT cells were consistent regardless of pretreatment with α1-2 fucosidase as a negative control (Fig. S6C and D). In contrast, the signal intensities of UEA-I in MDCK-PR8NA cells were higher than those in parental cells (Fig. S6A and E). Moreover, the signal intensity decreased by α1-2 fucosidase pretreatment (Fig. S6E and F), supporting the presence of α1-2 fucose on MDCK-PR8NA cells. Overall, the NA-expressing MDCK cells have the same glycome alterations of desialylation and glycan recapping with α1-2 fucose, which were observed in IAV-infected MDCK cells.

The mRNA expression of α1-2 fucosyltransferase genes in the NA-expressing MDCK cells was also evaluated by RT-qPCR to exclude the possibility of the increased expression of these genes (Fig. 4I). The results demonstrated that endogenous expression levels of FUT1 and FUT2 in MDCK-PR8NA cells were relatively lower, while those in MDCK-Vac2NA cells were slightly higher, compared to parental MDCK cells, with no significant difference. Accordingly, α1-2 fucosylation observed on the NA-expressing MDCK cells was not regulated by the FUT1 or FUT2 gene.

### Catalytic activity of NA is necessary for desialylation and glycan recapping with α1-2 fucose

NA expression in MDCK cells led to desialylation and glycan recapping, suggesting that the catalytic activity of NA is required for this glycan alteration. To test this hypothesis, catalytic-dead mutants of PR8 NA (E119V, D151G, and I222L) were expressed in MDCK cells (30–32), and the resulting bulk G418-resistant populations were subjected to lectin staining. As biologically independent NA-expressing cells showed variability in the signal intensities of SNA and UEA-I, the mean of three biological replicates was analyzed. Cells expressing wild-type PR8 NA (WT) exhibited lower SNA signal intensities than parental MDCK cells but higher intensities than the cloned MDCK-PR8NA cells (Fig. S7A and B). Both E119V and D151G mutant-expressing cells retained the SNA signal intensities, while the effect of the I222L mutation was limited (Fig. S7B and C). In contrast, UEA-I signal intensities in mutant NA-expressing cells were lower than those in WT-expressing cells (Fig. S7D). Specifically, E119V- and D151G-expressing cells exhibited much lower UEA-I signal intensities than I222L-expressing cells (Fig. S7E and F). Additionally, UEA-I signal and NA (detected by antiserum) merged in WT- and I222L-expressing cells, but not in E119V- or D151G-expressing cells (Fig. S7D), suggesting that the impairment of NA function by the E119V and D151G mutations contributed to the lower UEA-I signals in MDCK cells. Taken together, these results demonstrate that the catalytic activity of NA is required for desialylation and glycan recapping with α1-2 fucose in MDCK cells.

### Intracellular NA function is crucial for glycan recapping with α1-2 fucose in the IAV-infected cells

**Fig 5:**
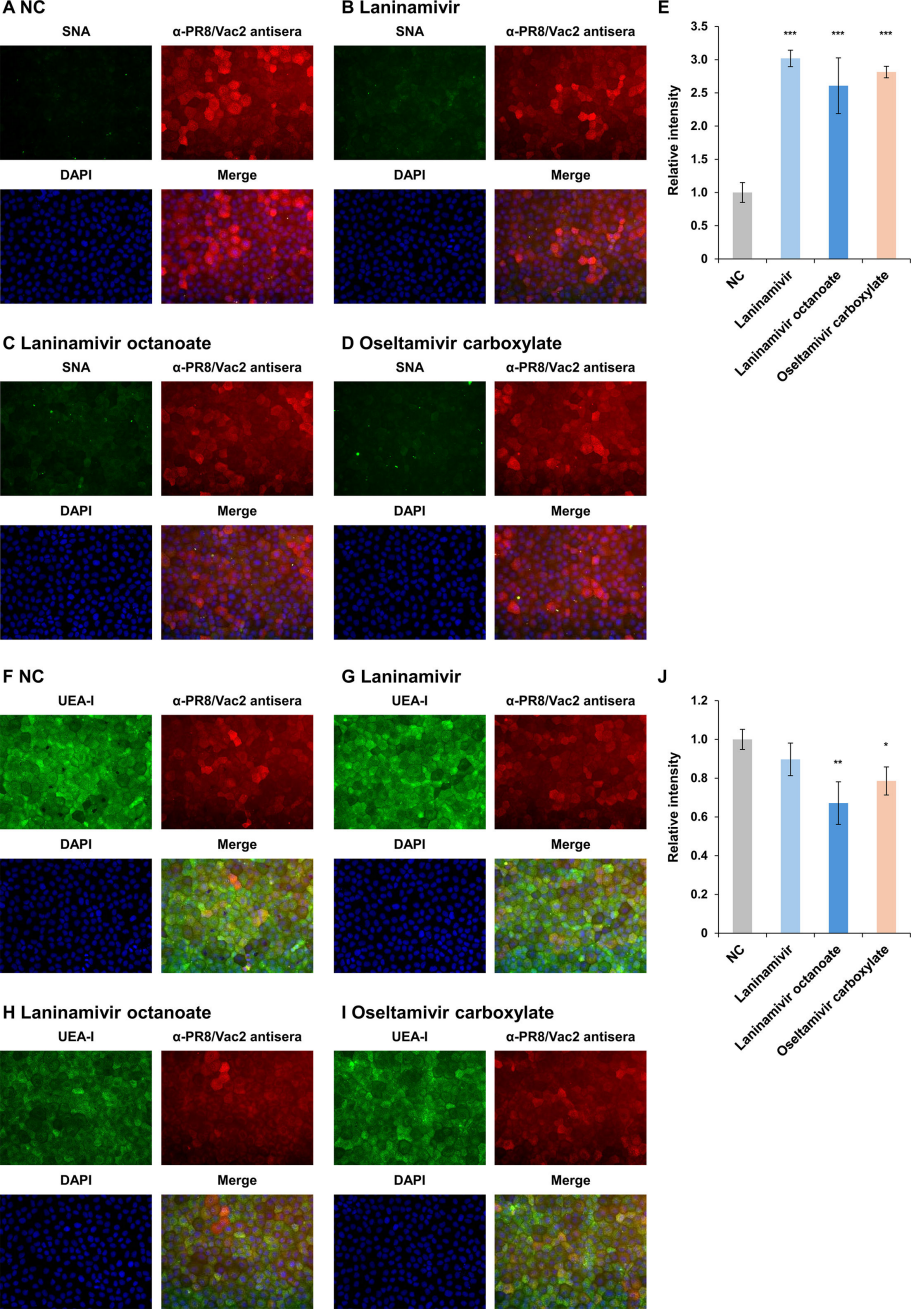
Glycan alterations induced by treatment with NA inhibitors in MDCK-PR8NA cells. (**A–D**) MDCK-PR8NA cells treated with NA inhibitor were stained with SNA. (**F–I**) MDCK-PR8NA cells treated with NA inhibitor were stained with UEA-I. Fluorescent intensities derived from SNA (**E**) and UEA-I (**J**) in an average of three fields of view were compared to non-treated control (NC). The statistical analysis was performed using the one-way ANOVA with Dunnett’s *post hoc* test (*, *P* < 0.05; **, *P* < 0.01; and ***, *P* < 0.001).

Since α1-2 fucosyltransferases are localized at the Golgi apparatus in host cells (23), glycan recapping with α1-2 fucose observed in NA-expressing and IAV-infected cells suggests that NA liberates sialic acids from the non-reducing terminus of glycans and serves LacNAc as an acceptor for α1-2 fucosyltransferases intracellularly. Thus, the focus of this study was to determine whether intracellular or extracellular functions of NA predominantly contribute to glycome alterations in cells. To inhibit extracellular NA activity in MDCK-PR8NA cells, cells were cultured in the presence of either of the three NA inhibitors, laninamivir, laninamivir octanoate, or oseltamivir carboxylate. Then, the reactivities of SNA and UEA-I were evaluated by lectin staining. The tests were performed with 200 µM of NA inhibitors, which inhibit the growth of PR8 virus in MDCK cells (Fig. S8A), and no influence of NA inhibitors on the increase in sialic acid or the decrease in α1-2 fucose was confirmed using MDCK-WT cells in advance (Fig. S8B through K). When the NA-expressing MDCK cells were treated with NA inhibitors, signal intensity of SNA was higher compared to the cells incubated without the inhibitor (Fig. 5A through E; Fig. S9A through E). The signal intensity of UEA-I remained high in laninamivir and oseltamivir carboxylate-treated cells, whereas the intensity decreased in laninamivir octanoate-treated cells (Fig. 5F through J; Fig. S9F through J). Since laninamivir octanoate can be taken up by cells and transformed into the active form within the cells (33), a decrease in the UEA-I signal intensity could reflect inhibition of NA function intracellularly. These results indicate that inhibition of extracellular NA activity did not influence the glycan recapping by α1-2 fucose in NA-expressing MDCK cells, while inhibition of intracellular NA activity led to a decrease in α1-2 fucose on the cells.

**Fig 6:**
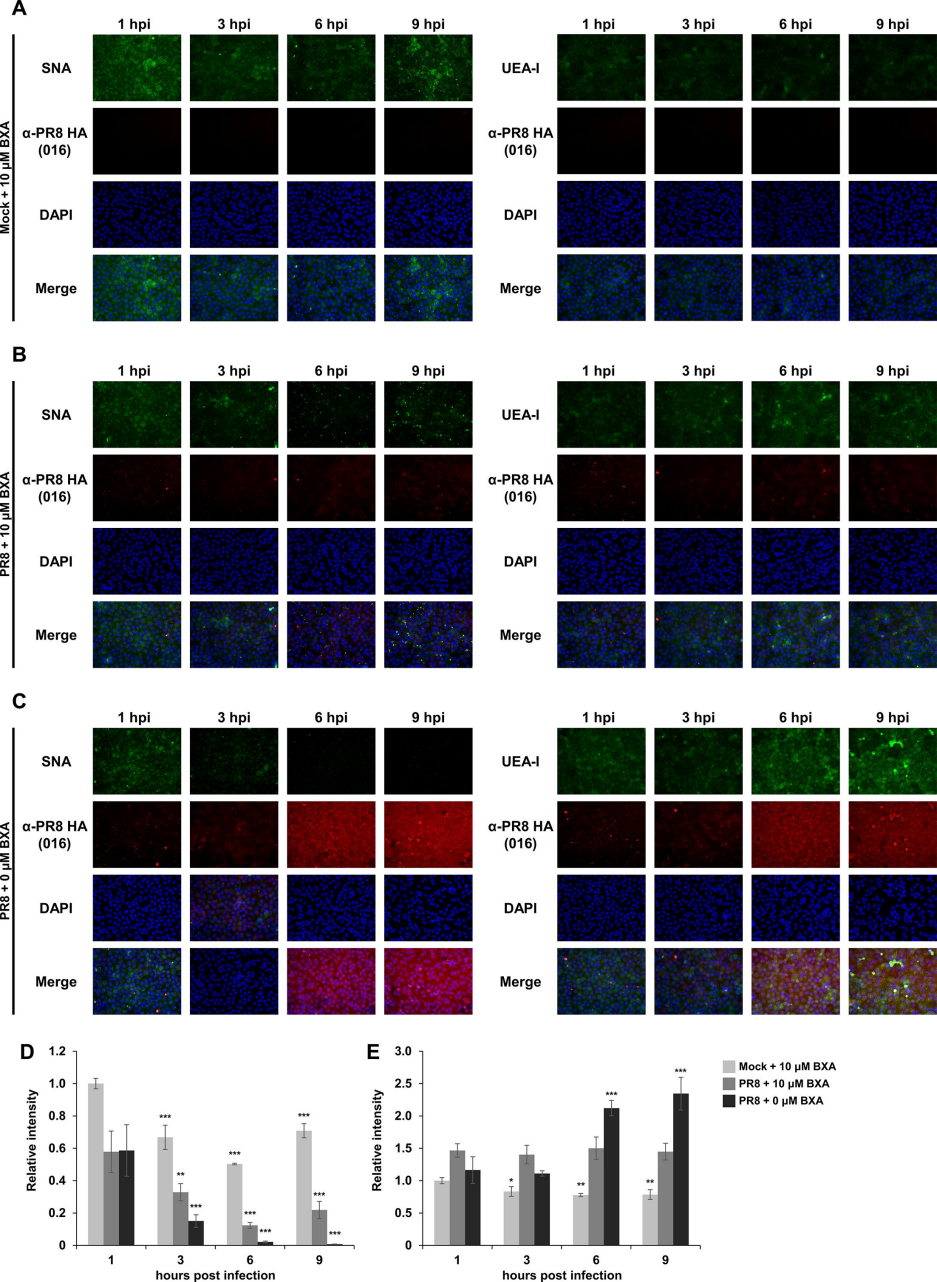
BXA treatment in the PR8-infected cells did not influence desialylation of glycans but inhibited the representation of α1-2 fucose. MDCK cells were infected with an extremely high titer of PR8 (MOI = 100) and cultured in the presence (**B**) or absence (**C**) of 1 μM BXA. The cells were fixed with 4% paraformaldehyde at multiple time points of 1, 3, or 6 hpi. The cells were then lectin stained with SNA or UEA-I (green) together with immunostaining with an anti-PR8 HA monoclonal antibody (red) and also counterstained with 4′,6-diamidino-2-phenylindole (DAPI) (blue). The signal intensities of each lectin in the PR8-infected cells were compared with those in the mock-infected cells (**A**). (**D**) Time-dependent fluorescent intensities derived from SNA in an average of three fields of view were compared. The data were represented by calculating relative intensities compared to 1 hpi of the mock-infected group. The statistical analysis was performed by one-way ANOVA with Dunnett’s *post hoc* test in comparison to 1 hpi as a control within a group (*, *P* < 0.05; **, *P* < 0.01; and ***, *P* < 0.001). (**E**) Time-dependent fluorescent intensities derived from UEA-I in an average of three fields of view were compared. The data were represented by calculating relative intensities compared to 1 hpi of the mock-infected group. The statistical analysis was performed by one-way ANOVA with Dunnett’s *post hoc* test in comparison to 1 hpi as a control within a group (*, *P* < 0.05; **, *P* < 0.01; and ***, *P* < 0.001).

To assess extracellular and intracellular NA functions in IAV-infected cells, MDCK cells were inoculated with an extremely high titer of PR8 (MOI = 100) premixed with BXA and stained with lectins. The signal intensity of SNA in the PR8-infected cells with or without BXA treatment decreased at 3 hpi (Fig. 6A through D). In particular, the cells cultured without BXA treatment showed a remarkable decrease in the SNA signal intensity at 6 and 9 hpi. On the other hand, an increase in UEA-I signal intensity was observed in the virus-infected cells cultured without BXA from 6 hpi, while the UEA-I signal remained low in the BXA-treated cells as well as the non-infected cells (Fig. 6C through E). To further assess the influence of extracellular NA activity on glycan alterations, MDCK cells were cultured with recombinant NA from *Vibrio cholerae* and subjected to lectin staining. The treatment with bacterial NA markedly decreased the signal intensity of SNA, whereas the UEA-I signal was not increased (Fig. S10). Consistent with the observations using NA-expressing cells, these results demonstrate that intracellular NA function is necessary for glycan recapping with α1-2 fucose. Conversely, NA on viral particles hydrolyzes sialylated glycans; however, extracellular NA functions cannot induce glycan recapping in IAV-infected cells.

### NA function contributes to superinfection exclusion in IAVs

**Fig 7:**
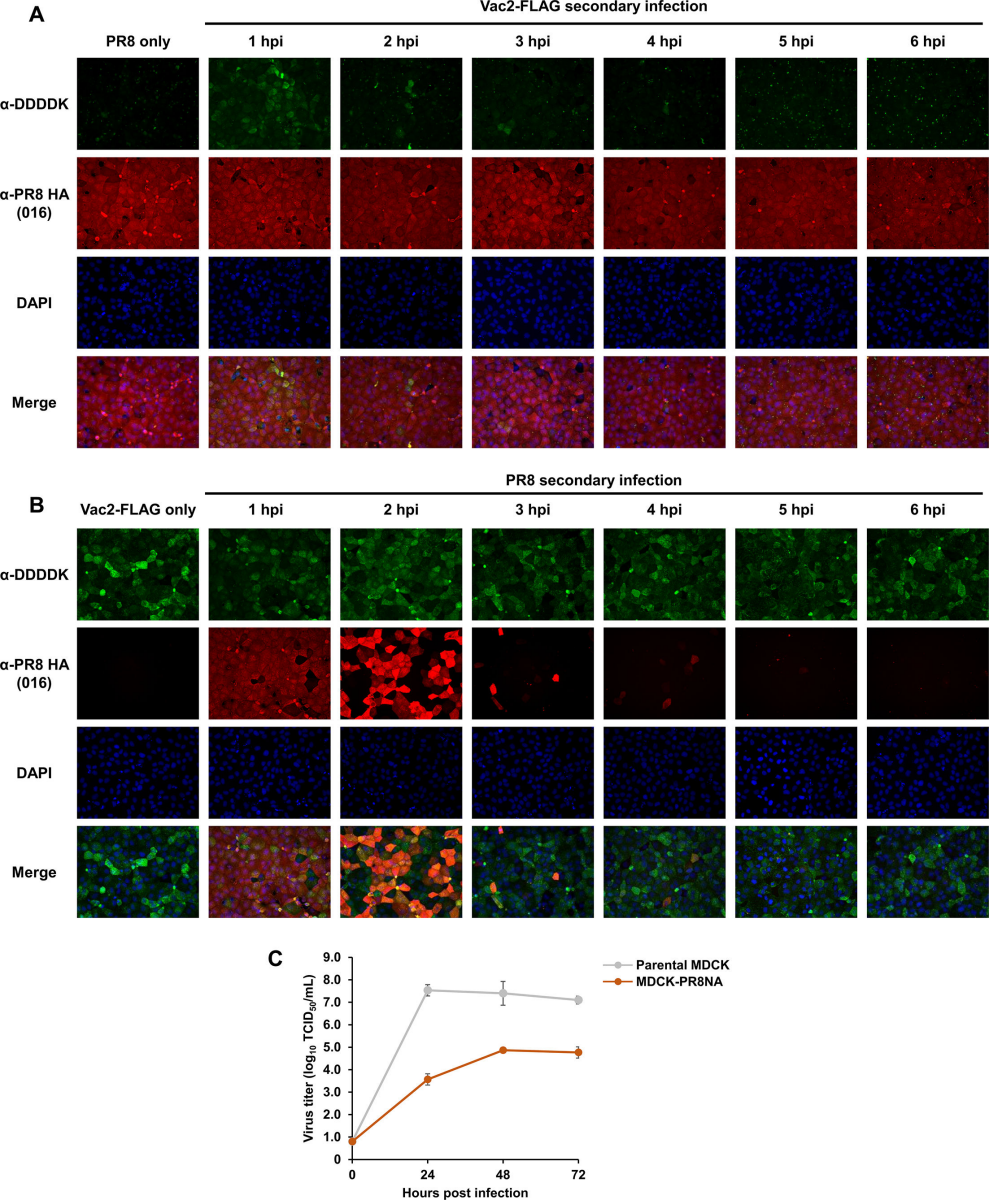
Superinfection exclusion was observed in MDCK cells infected with an IAV. (**A**) MDCK cells primarily infected with PR8 at MOI = 1 were secondarily inoculated with Vac2 at MOI = 1 at different time intervals. The cells were fixed in 4% paraformaldehyde at 8 hpi from the primary infection. Viral protein expression was detected by an anti-PR8 HA monoclonal antibody (red) or an anti-DDDDK monoclonal antibody for Vac2 NA (green) via immunofluorescent staining. The cells were counterstained with 4′,6-diamidino-2-phenylindole (DAPI) (blue). (**B**) MDCK cells primarily infected with Vac2 at MOI = 1 were secondarily inoculated with PR8 at MOI = 1 at different time intervals. The cells were fixed in 4% paraformaldehyde at 8 hpi from the primary infection. (**C**) Virus growth kinetics of PR8 in MDCK-PR8NA cells were compared with those in MDCK-WT cells. The culture supernatants in those cells were collected at each time point, and the infectious titer in the samples was calculated using MDCK cells.

**Fig 8:**
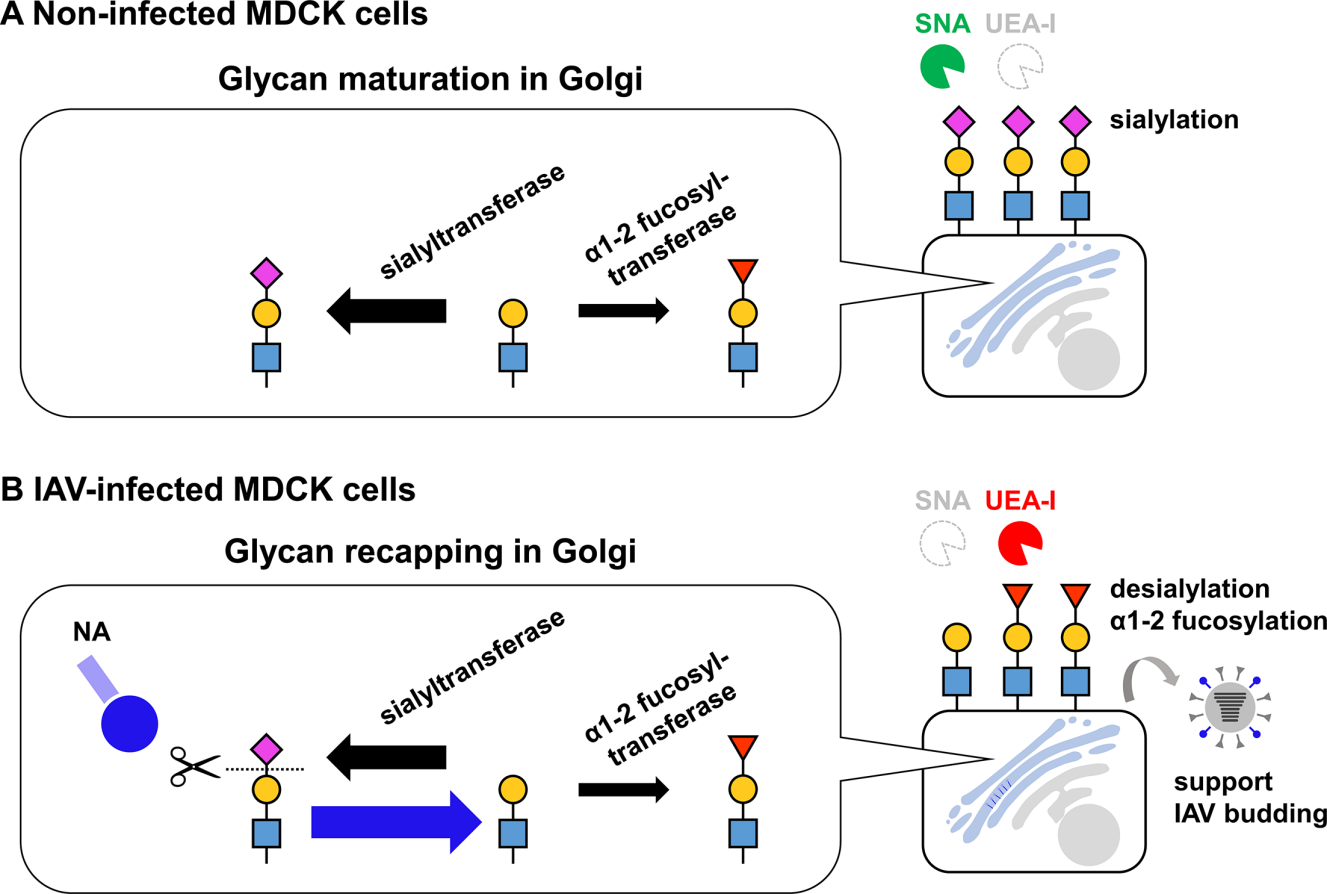
Glycome alterations observed in IAV-infected MDCK cells. (**A**) Sialylation of glycans is induced by sialyltransferases in the Golgi apparatus of non-infected MDCK cells, and terminal sialic acids displayed on the cell surface can be recognized by SNA. (**B**) Since NA obtains its function intracellularly during IAV infection, desialylation in the Golgi apparatus by the intracellular NA function induces glycan recapping with α1-2 fucose. The intracellular NA function blocks re-sialylation, contributing to efficient IAV budding.

Since sialic acid-containing glycans serve as initial receptors for IAVs on host cells, desialylation of IAV-infected cells by intracellular NA is likely to interfere with secondary IAV infection. To test this hypothesis, a superinfection experiment with IAVs was performed in MDCK cells at several time intervals between primary and secondary infections. MDCK cells infected with PR8 or Vac2-FLAG were inoculated with the other virus. After fixing the cells 8 h post-primary IAV infection, PR8 HA and Vac2 NA proteins were detected by immunofluorescent staining. The proportion of Vac2 NA-positive cells per PR8-primarily infected cell decreased when Vac2 was inoculated at a 2 h or longer interval after PR8 infection, and Vac2 NA-positive cells were not detected when there was a 5- or 6-h interval from primary PR8 infection to secondary Vac2 inoculation (Fig. 7A). The time intervals required for inhibition (2 h) and blocking superinfection (5 h) were similar, even when PR8 was inoculated into Vac2-primarily infected MDCK cells (Fig. 7B). To validate desialylation and α1-2 fucosylation on IAV-infected cells, PR8 or Vac2-FLAG virus was inoculated into MDCK cells under the same MOI and superinfection assay conditions, and the glycan profiles were monitored until 6 hpi by lectin staining and blotting (Fig. S11 to S13). SNA signal intensities in IAV-infected cells decreased from 3 hpi, and UEA-I signal intensities increased from 5 hpi (Fig. S11C and D and Fig. S12C and D). In addition, the NA expression in Vac2-FLAG-infected cells was confirmed slightly at 4 hpi and obviously at 5 hpi by western blotting, together with an increase in band intensities by lectin blotting with UEA-I (Fig. S13). Taking account of the impact of desialylation and α1-2 fucosylation on IAV-infected MDCK cells after 5 hpi (Fig. S11 to S13; Fig. 8), the complete superinfection exclusions by 5-h interval between two IAV infections were likely due to the lack of sialic acids on the cell surface.

The ability of PR8 to replicate in MDCK-PR8NA cells, which were established by cell cloning and expressed similar amounts of NA with IAV-infected cells at 5–6 hpi (Fig. S13), was also assessed. MDCK-PR8NA cells infected with PR8 did not exhibit any cytopathic effects for up to 72 hpi. In parallel, the viral titer in the supernatant was 10^4^-fold lower than that in parental MDCK cells at 24 hpi and remained lower until 72 hpi (Fig. 7C). These results suggested that NA expression in host cells restricted IAV infection.

## DISCUSSION

Multiple NA functions in the viral replication cycle, including cellular attachment, escape from decoy receptors, and release from infected cells, have been investigated in the context of those displayed on viral particles (5–9). Conversely, protein maturation and intracellular functions of NA are poorly understood, despite the potential of NA to act as a glycomic manipulator in host cells. The present study found that virus-infected cells displayed α1-2 fucose-capping glycans instead of sialylated ones, in agreement with previous studies (19, 20, 22). The global glycome alteration with desialylation and α1-2 fucosylation was also observed in NA-expressing MDCK cells without upregulation of α1-2 fucosyltransferase (FUT1 and FUT2) gene expressions, supporting the potential for intracellular NA function (Fig. 4). Since FUT1 and FUT2 were localized in the Golgi apparatus (23), the mechanisms of glycan recapping by α1-2 fucose observed in IAV-infected cells can be explained by intracellular NA functions: viral NA has obtained its catalytic activity in the Golgi apparatus and provides acceptors for α1-2 fucosyltransferases as a competitor of sialyltransferases (Fig. 8). Moreover, the occupation of the glycan terminus by α1-2 fucose as a result of intracellular NA functions (Fig. 1 and 2) limits the opportunities of re-sialylation. Hence, in addition to the conventional theory that NA functions cleave sialic acids on the cellular surface during viral release, intracellular NA functions provide an advantage for virus release by reducing sialoglycan traps. These findings highlighted a novel mechanism of NA action that supports viral budding in IAV-infected cells. Additionally, only an NA inhibitor, laninamivir octanoate, had the potential to block intracellular NA activity (Fig. 5; Fig. S9), suggesting that low intracellular accumulation of conventional NA inhibitors is challenging to inhibit the intracellular NA function (34). Thus, understanding the mechanism of action of NA may accelerate the development of antivirals that target NA.

Glycan capping with sialic acids in host cells is regulated by β-galactoside α2,3-sialyltransferases (ST3Gals) and α2,6-sialyltransferases (ST6Gals) (35). A previous study revealed that knocking out either ST3Gals or ST6Gals (KO) in MDCK cells increased the proportion of Siaα2,6Gal in ST3Gals-KO cells and Siaα2,3Gal in ST6Gals-KO cells (36). In contrast, double KO cells, as well as sialic acid transporter (SLC35A1)-KO cells, showed a lack of sialylated glycans. Corresponding to the global desialylation, these cells reacted remarkably strongly to UEA-I, TJA-II, and EEL in the lectin microarray. Taken together with glycan recapping observed with IAV-infected cells in this study, these observations indicate that intracellular global desialylation is a trigger of glycan recapping with α1-2 fucose and/or α1-3 galactose in MDCK cells.

The structure of α1-2 fucose, which is known as the H antigen, is commonly observed in the ABO blood group antigens and is synthesized by FUT1, expressed in erythroid precursors (37). In contrast, FUT2 is a secretor transferase that synthesizes H antigen in epithelial tissues and salivary glands (38). Interactions of α1-2 fucose motifs with microorganisms have been extensively reported. Norwalk virus and rotavirus recognize them for viral attachment (39, 40), and some bacteria on intestinal epithelia regulate α1-2 fucosylation via cytokine signals (41). Although α1-2 fucose on the IAV-infected cells is likely to be a consequence of intracellular NA activity, the unique glycan structure may have an unknown role in differentiating IAV-infected cells from non-infected ones. It is of interest that glycan recapping by α1-3 galactose was not detected in this study, dissimilar from previous studies (19, 20, 22). The MDCK cells originating from canine possess the functional α1-3 galactosyltransferase gene required for synthesizing α1-3 galactose at the non-reducing terminus of glycans (42). Since the monosaccharides used in the enzymatic glycosylation rely heavily on the sugar intake of cells (43), differences in culture conditions or components of the cell culture medium, such as fetal bovine serum, might influence the glycome of IAV-infected cells.

Lectin staining of A549 and Vero E6 cells revealed desialylation following IAV infection (Fig. S3). Additionally, lectin staining of respiratory tissues from IAV-infected animals showed no binding of recombinant HA in their epithelia, especially in the cells that were positive for IAV antigens (44), suggesting desialylation by the NA function following IAV infection. In contrast, α1-2 fucosylation could not be detected in other cell lines except MDCK cells. Consistent with this observation, virus particles propagated in A549 and Vero E6 cells have strongly reacted with chitin-binding lectins, suggesting higher representations of poly-LacNAc structures rather than recapping with α1-2 fucose (20). These observations suggested that intracellular NA functions contribute to the desialylation in the IAV-infected cells, while the glycan recapping process is dependent on the glycan synthesis machinery in the host cells. The present study could assess the intracellular NA functions by detecting α1-2 fucose on the cellular surface, because the α1-2 fucosylation proceeds exclusively on glycan capping with sialic acid in the Golgi of host cells. Accordingly, differentiation of intracellular and extracellular NA functions in the IAV-infected cells is still challenging without the unique glycan structures exclusively present on the glycan terminus. Accordingly, the present study could not prove the intracellular NA activity in other cell lines except MDCK cells.

In agreement with low PR8 replication in the NA-expressing cells in this study, the sialic acid transporter SLC35A1-knockout MDCK cells or sialyltransferase (all ST3Gal and ST6Gal series)-deficient MDCK cells exhibited significantly decreased IAV replication ability (36). Since the intracellular NA activity in NA-expressing cells competes for sialyltransferases and induces a similar glycome as the SLC35A1-knockout or sialyltransferase-deficient cells, low IAV growth in these cells suggests that sialic acids on the cell surface are required for efficient IAV replication but are not necessary for infection. These observations correspond to the theory that functional receptors, such as epidermal growth factor (EGFR) or calcium channels (CaV1.2), are required for IAV entry into cells via clathrin-mediated endocytosis or micropinocytosis, although IAVs use sialosides for cell attachment (45–47). Indeed, the absence of sialic acids in cells reduced interactions between IAVs and EGFR, inducing fewer downstream signals and clathrin-mediated endocytosis (48). Thus, global desialylation of IAV-infected cells could reduce the opportunities for secondary infecting viruses to access functional receptors, thereby potentially shaping the window for superinfection.

While the timeframe of superinfection exclusion of IAVs differs among strains, cell types, and experimental settings, opportunities for secondary IAV infections are less frequent, as the duration from primary infection is longer (10, 11, 49). The early stage of superinfection exclusion at 2–5 h after primary infection can be induced by impairing nuclear import of the genome of the secondary infected virus, even if the virus can enter the cell (50). In contrast, an altered glycan repertoire with desialylation and α1-2 fucosylation mediated by intracellular NA function on IAV-infected cells at 5 h post-primary infection restricts the entry of secondary virus. The mechanism, in combination with the liberation of sialic acid by extracellular NA expressed on the plasma membrane, facilitates the latter phase of superinfection exclusion. Moreover, the short window for superinfection in advancing budding may be beneficial for IAVs to facilitate a viral replication cycle using sufficient intracellular resources and produce progeny virions at the cellular level.

In conclusion, this study indicated that the NA protein obtains its catalytic function intracellularly and induces glycome alterations in infected MDCK cells. The intracellular NA function contributes to global desialylation and glycan recapping with α1-2 fucose, leading to efficient virus release from host cells. This demonstrated a novel theory that intracellular NA activity, in addition to extracellular activity, hydrolyzes sialosides and blocks interactions between progeny virus particles and the virus receptor to advance virus budding. Consequently, glycan recapping induced by the intracellular NA functions potentially restricts secondary IAV infection in cells, resulting in a strict window for superinfection. These findings also provide insights into the evolutionary strategies of IAVs by maintaining a balance between successful replication and reassortment under limited superinfection opportunities controlled by the NA functions.

## MATERIALS AND METHODS

### Viruses and cells

The IAVs A/Puerto Rico/8/1934 (H1N1) (PR8), A/duck/Mongolia/47/2001 (H7N1), A/duck/Mongolia/54/2001 (H5N2), A/duck/Hokkaido/84/2002 (H5N3), A/duck/Hokkaido/95/1981 (H8N4), A/duck/Hokkaido/66/2001 (H12N5), A/duck/England/1/1956 (H11N6), A/duck/Ukraine/1/1963 (H3N8), and A/duck/Hokkaido/W245/2004 (H11N9) were used. A recombinant IAV, Vac2/P0NA-FLAG (Vac2-FLAG), which was previously generated via reverse genetics from a plasmid encoding FLAG-tagged NA with the backbone of A/duck/Hokkaido/Vac-2/2004 (H7N7) (51), was used. Viruses were propagated in 10-day-old embryonated chicken eggs at 35°C for 48 h, and these infectious allantoic fluids were stored at –80°C until use as virus stocks.

MDCK and MDCK-FUT cells (29) were maintained in minimum essential medium (MEM; Shimadzu Diagnostics Corporation, Tokyo, Japan) supplemented with 0.3 mg/mL L-glutamine (Nacalai Tesque Inc., Kyoto, Japan), 100 U/mL penicillin G (Meiji Seika Pharma Co., Ltd., Tokyo, Japan), 0.1 mg/mL streptomycin (Meiji Seika Pharma), 8 μg/mL gentamicin (Takata Pharmaceutical Co., Ltd., Saitama, Japan), and 10% fetal bovine serum (Merck KGaA, Darmstadt, Germany) in an incubator at 37°C with 5% CO_2_. A549 and Vero E6 cells were maintained in DMEM (Thermo Fisher Scientific, Waltham, MA, USA) supplemented with 0.3 mg/mL L-glutamine (Nacalai Tesque), 100 U/mL penicillin G (Meiji Seika Pharma), 0.1 mg/mL streptomycin (Meiji Seika Pharma), 8 μg/mL gentamicin (Takata Pharmaceutical), and 10% fetal bovine serum (Nichirei Biosciences Inc., Tokyo, Japan) in an incubator at 37°C with 5% CO_2_.

### Antibodies and lectins

Anti-PR8 HA monoclonal antibodies (Clones: 2F1A7 and 016) were purchased from Sino Biological (11684-MM03 and 11684-R016; Beijing, China), and biotinylated anti-DDDDK-tag monoclonal antibody (Clone: FLA-1) was purchased from Medical & Biological Laboratories (M185-6; Tokyo, Japan). The HA antibody was purified using a MonoSpin ProG column (GL Sciences, Tokyo, Japan) and then biotinylated using a Biotin Labeling Kit-NH_2_ (Dojindo Laboratories, Kumamoto, Japan) for immunoprecipitation of PR8 HA, as previously described by Hiono et al. (20). For the detection of HA derived from AIVs, H5 and H7 HA monoclonal antibodies previously established (64/1 has been described by Soda et al. [52] and 253/1 has been described by Manzoor et al. [53], respectively) were used. In addition, H3 HA monoclonal antibody (D17/4) established using A/duck/Hokkaido/8/1980 (H3N2), H8 HA monoclonal antibody (66/B8) established with A/turkey/Ontario/6118/1968 (H8N4), H11 HA monoclonal antibody (1C6/2/1) established with A/duck/England/1/1956 (H11N6), and H12 HA monoclonal antibody (146/5/1) established with A/duck/Alberta/60/1976 (H12N5) were kindly provided by Prof. Ayato Takada. An anti-NP monoclonal antibody (Clone: 183/5) was previously established using A/swine/Hong Kong/10/1998 (H9N2) (44). The anti-sialyl Lewis X antibody (Clone: KM93) was purchased from Kyowa Kirin (Tokyo, Japan), and the anti-β-actin monoclonal antibody (Clone: BA3R) was purchased from Applied Biological Materials (Richmond, BC, Canada). Fluorescein-conjugated SNA (FL-1301-2) and biotinylated UEA-I (B-1065-2) were purchased from Vector Laboratories (Burlingame, CA, USA). Fluorescein-conjugated ECA was purchased from EY Laboratories (F-5901-5; San Mateo, CA, USA). Two fluorescently labeled streptavidin-Alexa Fluor 488 and 555 conjugates were purchased from Thermo Fisher Scientific, and horseradish peroxidase (HRP)-conjugated streptavidin was purchased from Jackson ImmunoResearch (West Grove, PA, USA). Chicken polyclonal antisera against PR8 or Vac2 were also used for immunostaining of NA-overexpressing cells. The secondary antibodies used were anti-mouse IgG-Alexa Fluor 568, anti-rabbit IgG-Alexa Fluor 555, anti-rabbit IgG-Alexa Fluor 594 (Thermo Fisher Scientific), anti-chicken IgG-TRITC (Abnova, Taipei, Taiwan), anti-mouse IgG-FITC (Rockland Immunochemicals Inc., Pottstown, PA, USA), anti-mouse IgG-HRP (Bio-Rad Laboratories Inc., Hercules, CA, USA), and anti-FITC-HRP (Southern Biotech, Birmingham, AL, USA).

### Virus titration

Ten-fold dilutions of the virus stocks and cellular supernatants were inoculated into confluent monolayers of MDCK cells and incubated at 35°C for 1 h. The viral solution, including unbound viruses, was removed from the cells, and the cells were washed with serum-free MEM. Cells were cultured in serum-free MEM containing 1.0 μg/mL of acetylated trypsin (Merck) at 35°C for 72 h. The infectious titers of the viruses were expressed as 50% tissue culture infectious dose (TCID_50_), determined using the method reported by Reed and Muench (54) by observing the cytopathic effect in virus-infected cells.

### Assessment of the inhibitory effects of NA inhibitors on virus growth

Confluent monolayers of MDCK cells seeded in 24-well plates were inoculated with PR8 at 100 TCID_50_/well and incubated at 35°C for 1 h. The viral solution, including unbound viruses, was removed from the cells, and the cells were washed with serum-free MEM. The cells were then cultured in serum-free MEM containing 1.0 μg/mL of acetylated trypsin (Merck) and the corresponding dose of each NA inhibitor, laninamivir (MedChemExpress, Monmouth Junction, NJ, USA), laninamivir octanoate (Merck), and oseltamivir carboxylate (ChemScene, Monmouth Junction, NJ, USA), at 35°C for 48 h. Culture supernatants were collected, and the virus titers were determined.

### Cloning of NA expression plasmids

The NA gene of PR8 was amplified and independently cloned into the pGEM-T Easy Vector (Promega Corp., Madison, WI, USA) by ligation with T4 DNA Ligase (Promega Corp.). A nucleotide sequence encoding a FLAG (DYKDDDDK) tag with a single glycine linker (G) and stop codon was inserted into the C-terminal coding region of each NA gene using the KOD Plus Mutagenesis Kit (Toyobo, Osaka, Japan), and the construct was designated pGEM-T-PR8NA-FLAG. The pGEM-T-PR8NA-FLAG and pCXN2-empty vectors were digested with *Eco*RI (Toyobo) at 37°C for 1 h, and the NA-coding fragments were cloned into the pCXN2 expression vector (55, 56) using the DNA Ligation kit Mighty Mix (Takara Bio Inc., Shiga, Japan) and designated as pCXN2-PR8NA-FLAG. The Vac2NA-coding fragments were amplified from pHW2000-Vac2NA-FLAG, which was previously constructed to rescue an infectious virus (51), and cloned into the pCXN2 vector (pCXN2-Vac2NA-FLAG) using an In-Fusion HD Cloning Kit (Takara Bio Inc.).

To generate the catalytic-dead NA-coding plasmids, a nucleotide mutation encoding an amino acid substitution of E119V (GAG to GTG) (30), D151G (GAC to GGC) (31), and I222L (ATA to CTA) (32) in PR8 NA was individually introduced into pCXN2-PR8NA-FLAG. The mutations were introduced by site-directed mutagenesis using KOD One DNA polymerase (Toyobo) and complementary primer sets for each substitution.

### Establishment of NA stably overexpressed MDCK cells

MDCK cells cultured on a 12-well plate were transfected with 1 μg of pCXN2-PR8NA-FLAG or pCXN2-Vac2NA-FLAG using Lipofectamine 3000 (Thermo Fisher Scientific) and maintained in a culture medium with 500 μg/mL of G418 sulfate (FUJIFILM Wako Pure Chemical Corp., Osaka, Japan). To establish cloned NA-expressing cells, the transfected cells were passaged seven times, and G418-resistant clones were isolated and transferred to 12-well plates. Each clone was passaged four times in the presence of G418 sulfate and frozen to prepare the cell stock.

For the establishment of catalytic-dead NA-expressing cells, 1 μg of pCXN2-PR8NA-FLAG or its mutants was mixed with Lipofectamine 3000 (Thermo Fisher Scientific) in a 12-well plate. A suspension of MDCK cells was then added to the plate and incubated in Opti-MEM (Thermo Fisher Scientific) at 37°C for 48 h in the presence of two TBK1 inhibitors, 1 μM of BX795 (Merck) and 1.5 μM of amlexanox (Tokyo Chemical Industry, Tokyo, Japan) (57). The cells were maintained in culture medium containing 500 μg/mL of G418 sulfate (FUJIFILM Wako Pure Chemical Corp.). The resulting G418-resistant cell populations were passaged twice and subjected to lectin staining.

### Lectin and immunofluorescent staining

Confluent monolayers of MDCK, MDCK-FUT, MDCK-PR8NA, and MDCK-Vac2NA cells were seeded on glass chamber slides (Matsunami Glass, Osaka, Japan), washed with phosphate-buffered saline (PBS), and fixed for 10 min in cold methanol or 4% paraformaldehyde in PBS. For staining of IAV-infected cells, MDCK cells were infected with either PR8 or Vac2 at an MOI of 0.1, 1, or 100, and then fixed. For functional analyses of NA, virus-infected MDCK cells were maintained in serum-free MEM supplemented with acetylated trypsin, laninamivir (MedChemExpress), laninamivir octanoate (Merck), oseltamivir carboxylate (ChemScene), or BXA (Shionogi, Osaka, Japan). For the assessment of extracellular NA function, confluent monolayers of MDCK cells were cultured in serum-free MEM supplemented with equivalent units of NA from *Vibrio cholerae* (Merck, N7885) at 37°C for 16 h. Before staining, the cells were washed three times with PBS. To detect viral antigens and glycan epitopes, the cells were incubated with the corresponding primary antibody (anti-NP, anti-PR8 HA, or anti-DDDDK monoclonal antibody) together with lectin (FITC-SNA or biotinylated UEA-I) at 4°C overnight. After washing three times with PBS, the cells were incubated with the appropriate fluorescent-labeled secondary antibodies or streptavidin at room temperature for 2 h. After washing three times with PBS, the cells were counterstained with 4′,6-diamidino-2-phenylindole (Dojindo Laboratories, Kumamoto, Japan). The cells were mounted using the SlowFade Diamond Antifade Mountant (Thermo Fisher Scientific) and observed under a fluorescence microscope (Axiovert 200; Carl Zeiss, Oberkochen, Germany). The signal intensities of fluorescent images staining with lectins were quantitated by ImageJ software version 1.54g (58). The data are represented as mean signals of three fields of view ± standard deviations (SDs). The mean of signal intensities was statistically analyzed by Student’s *t*-test or one-way ANOVA with Dunnett’s *post hoc* test via EZR software version 1.63 (59).

### Lectin microarray analysis

Lectin microarray analyses were performed as previously described with some modifications (20, 25, 36). Briefly, for virus-infected cells, confluent MDCK cells were inoculated with PR8 at an MOI = 1 or PBS as a mock and cultured at 35°C for 1 h. The cells were then washed and cultured in serum-free MEM containing 1.0 μg/mL of acetylated trypsin at 35°C for 12 h. The IAV-infected cells and NA-expressing cells were washed with ice-cold PBS three times and labeled with 1 mg/mL of EZ-Link Sulfo-NHS-Biotin (Thermo Fisher Scientific) at 4°C for 1 h. The cells were then washed with Tris-buffered saline (TBS) to quench the remaining biotin-labeling reagents and lysed with TBS containing 1% Nonidet P-40, 0.5% sodium deoxycholate, 0.1% SDS, and an EDTA-free protease inhibitor (cOmplete ULTRA Tablets, Mini, EDTA-free; Roche, Basel, Switzerland). Protein samples were diluted with probing buffer (TBS containing 1% Triton X-100, 500 mM glycine, 1 mM CaCl_2_, and 1 mM MnCl_2_) and applied to an activated LecChip (version 1.0; Precision System Science Co., Ltd., Chiba, Japan; Table S1). The array glass slides were incubated overnight at 20°C. After washing, slides were overlaid with streptavidin and Alexa Fluor 555 conjugate (Thermo Fisher Scientific). After washing, the slides were scanned using an evanescent field excitation fluorescence imager (GlycoStation Reader 1200, Precision System Science). Luminance obtained from the images was measured using GlycoStation ToolsPro Suite version 2 (Precision System Science). The data are mean-normalized and represented as mean signals of three spots ± SD.

### Immunoprecipitation

Confluent MDCK cells were inoculated with PR8 at an MOI = 1 or PBS as a mock and cultured at 35°C for 1 h. Cells were washed and cultured in serum-free MEM containing 1.0 μg/mL of acetylated trypsin at 35°C for 24 h. Then, 1 mL of infectious cell culture supernatants was mixed with trichloroacetic acid (TCA, FUJIFILM Wako Pure Chemical Corp.) and incubated at 4°C for 3 h to precipitate proteins in the cellular supernatant. After centrifugation, TCA was removed, and the pellet was washed with cold acetone. The protein samples were centrifuged, and the pellet was resolved in 1 M Tris-HCl (pH 8.6) containing 1% SDS and 1.5% 2-mercaptoethanol. The samples were subjected to sodium dodecyl sulfate-polyacrylamide gel electrophoresis, western blotting, and lectin blotting of the cell supernatant (Sup). The original cellular supernatant was also immunoprecipitated to enrich HA proteins as immunoprecipitated samples. Biotinylated anti-HA monoclonal antibodies (200 ng; Sino Biological) were added, and after incubation at 37°C for 1 h, the complexes were captured with Dynabeads MyOne Streptavidin T1 (Thermo Fisher Scientific) at 4°C for 1 h. After washing, viral antigens were eluted from the magnetic beads by disruption with 100 mM citric acid containing 1% Triton X-100. Biotinylated antibodies that contaminated the eluted samples were depleted by incubating them with Dynabeads MyOne Streptavidin T1 at 4°C for 1 h. The culture supernatant of mock-infected cells was used as a negative control.

### SDS-PAGE, silver staining, and western and lectin blotting

Samples were mixed with an equivalent amount of 2× Laemmli sample buffer (125 mM Tris-HCl [pH 6.8], 4% SDS, 20% glycerol, 0.02% bromophenol blue, and 10% 2-mercaptoethanol). SDS-PAGE was conducted using a 4–15% Mini-PROTEAN TGX Precast Protein Gel (Bio-Rad) at a constant voltage of 200 V for 35 min under reducing conditions. For silver staining, the proteins on the gel were stained with EzStain Silver (AE-1360; Atto Corp., Tokyo, Japan) following the commercial protocol. For western and lectin blotting, the resolved proteins were transferred to Immobilon-P PVDF membranes (Merck). The membrane was blocked with a PVDF Blocking Reagent for Can Get Signal (Toyobo). To detect viral HA or NA, the membrane was probed with an anti-PR8 HA monoclonal antibody (2F1A7, 1:2,000 dilution; Sino Biological) or an anti-DDDDK tag monoclonal antibody (FLA-1, 1:2,000 dilution; Medical & Biological Laboratories) and then with a goat anti-mouse IgG monoclonal antibody conjugated with HRP (1:10,000 dilution; Bio-Rad). For loading control, the membrane was probed with an anti-β-actin monoclonal antibody (BA3R, 1:2,000 dilution; Applied Biological Materials), followed by the mouse-IgG HRP conjugate (1:10,000 dilution). For lectin blotting, the membrane was probed with FITC-labeled ECA (1:2,000 dilution; EY Laboratories) or biotinylated UEA-I (1:2,000 dilution; Vector Laboratories) or FITC-labeled SNA (1:2,000 dilution; Vector Laboratories), and appropriate HRP-conjugates, goat anti-FITC antibody (1:10,000 dilution; Southern Biotech), or HRP-streptavidin (1:10,000 dilution; Thermo Fisher Scientific), respectively. Proteins were detected using a chemiluminescent substrate, ImmunoStar LD (FUJIFILM Wako Pure Chemical Corp.), or Immobilon Crescendo Western HRP substrate (Merck) and visualized using LuminoGraph I (WSE-6100, Atto Corp.).

### RT-qPCR of glycosyltransferases

Lysates of PR8-infected cells and NA-expressing cells were collected and subjected to quantification of fucosyltransferase (FUT1 and FUT2) genes, with the glyceraldehyde-3-phosphate dehydrogenase (GAPDH) gene as a housekeeping gene. Total RNA was extracted from cellular lysates using TRIzol Reagent (Thermo Fisher Scientific) in triplicate and reverse-transcribed into cDNA using ReverTra Ace (Toyobo). Subsequently, qPCR was performed with the KOD SYBR qPCR Mix (Toyobo) using the Applied Biosystems StepOnePlus real-time PCR system (Thermo Fisher Scientific). The primer sequences used for qPCR are listed in Table S2 (60). Specific amplification of the fucosyltransferase and GAPDH genes was confirmed using melting curve analysis. The relative mRNA expression levels of target genes were calculated by the comparative Ct method and presented as 2^−ΔΔCt^, with a housekeeping gene encoding GAPDH as an internal control. The data are represented as the mean expression level of biological triplicates ± SD.

### Assessment of superinfection exclusion

Confluent monolayers of MDCK cells seeded on glass chamber slides (Matsunami Glass) were inoculated with either PR8 or Vac2-FLAG at an MOI of 1 and incubated at 35°C for 30 min. The viral solution, including unbound viruses, was removed from the cells, and the cells were washed with serum-free MEM. The cells were then cultured in serum-free MEM containing 1.0 μg/mL of acetylated trypsin (Merck) at 35°C until the secondary infection. After washing with serum-free MEM, PR8 or Vac2-FLAG was inoculated into the cells at an MOI of 1 and further incubated at 35°C for 30 min. The viral solution was removed from the cells, and the cells were washed once with serum-free MEM. The cells were then cultured in serum-free MEM containing 1.0 μg/mL of acetylated trypsin (Merck) at 35°C until 8 h post-primary viral infection. The cells were then washed with PBS and fixed with 4% paraformaldehyde in PBS at room temperature for 10 min. Viral antigens on the cells were stained using the abovementioned immunofluorescence staining method.

## Data Availability

The data underlying this study are available in the article and supplemental material.

## SUPPLEMENTAL MATERIAL

Supplemental figures spectrum.03328-25-s0001.pdf Fig. S1 to S13.

Supplemental material spectrum.03328-25-s0002.docx Supplemental figure legends.

Table S1 spectrum.03328-25-s0003.xlsx List of 45 lectins used in lectin microarray analyses.

Table S2 spectrum.03328-25-s0004.xlsx Primer list used for RT-qPCR of glycosyltransferase genes.

ASM does not own the copyrights to Supplemental Material that may be linked to, or accessed through, an article. The authors have granted ASM a non-exclusive, world-wide license to publish the Supplemental Material files. Please contact the corresponding author directly for reuse.
