# Supplementary material for: Neuraminidase of influenza A viruses induces global desialylation of host cells via its intracellular function
Source: Microbiol Spectr. 2026 Feb 18;14(4):e03328-25. doi: 10.1128/spectrum.03328-25 (PMC13055363; doi:10.1128/spectrum.03328-25)
Supplement: Supplemental figures — Fig. S1 to S13. [file spectrum.03328-25-s0001.pdf]

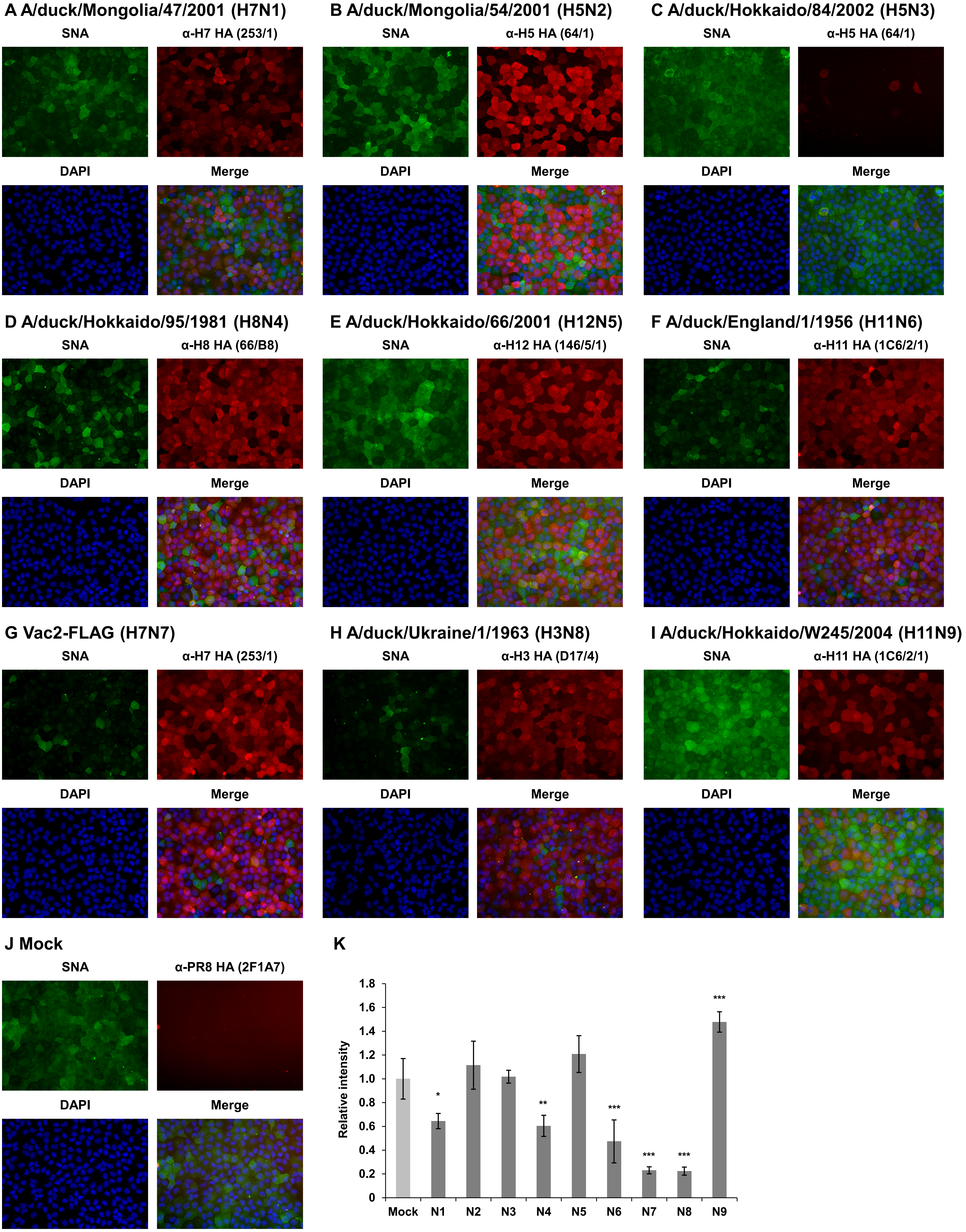

Fig S1

Kobayashi *et al*

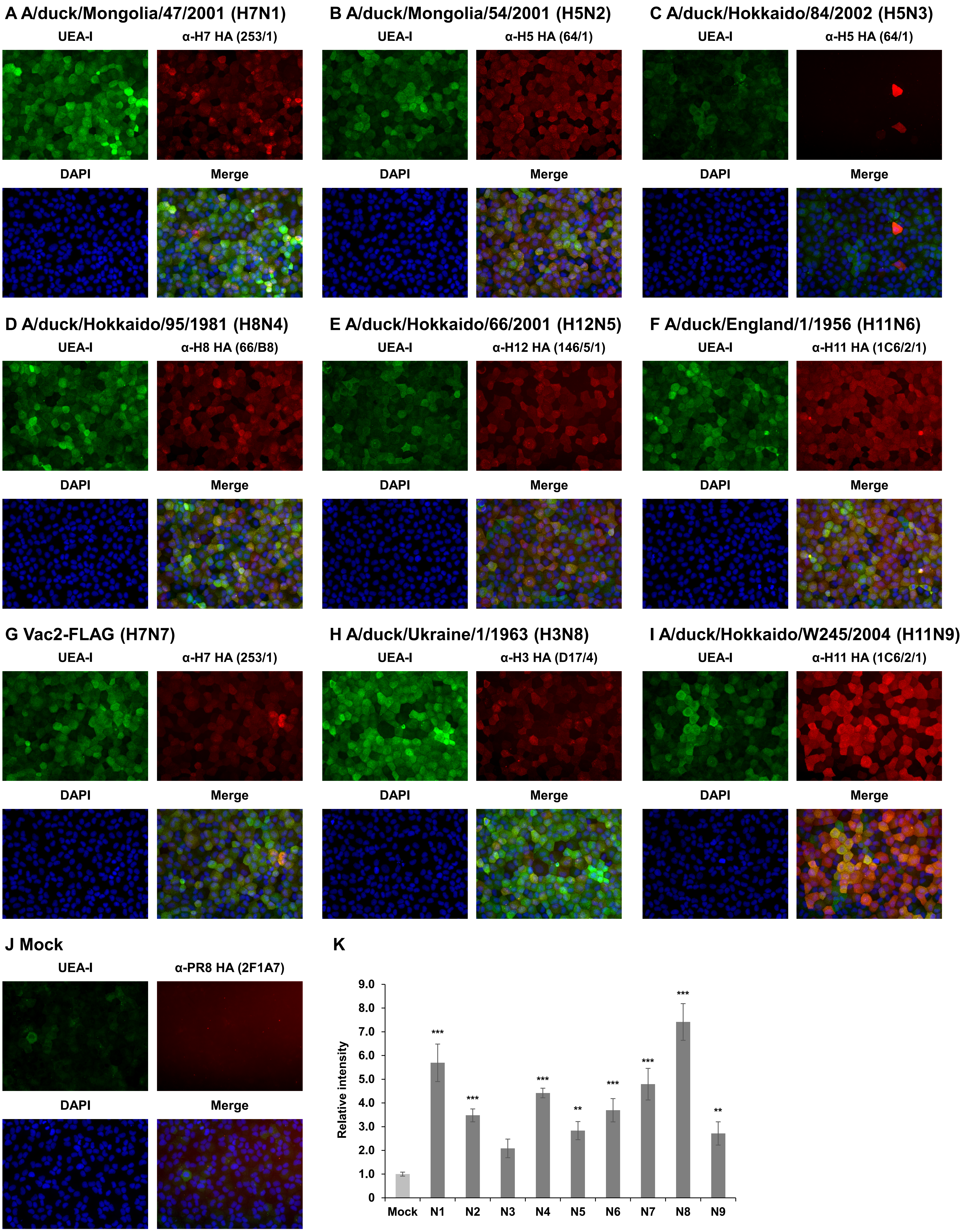

Fig S2

Kobayashi *et al*

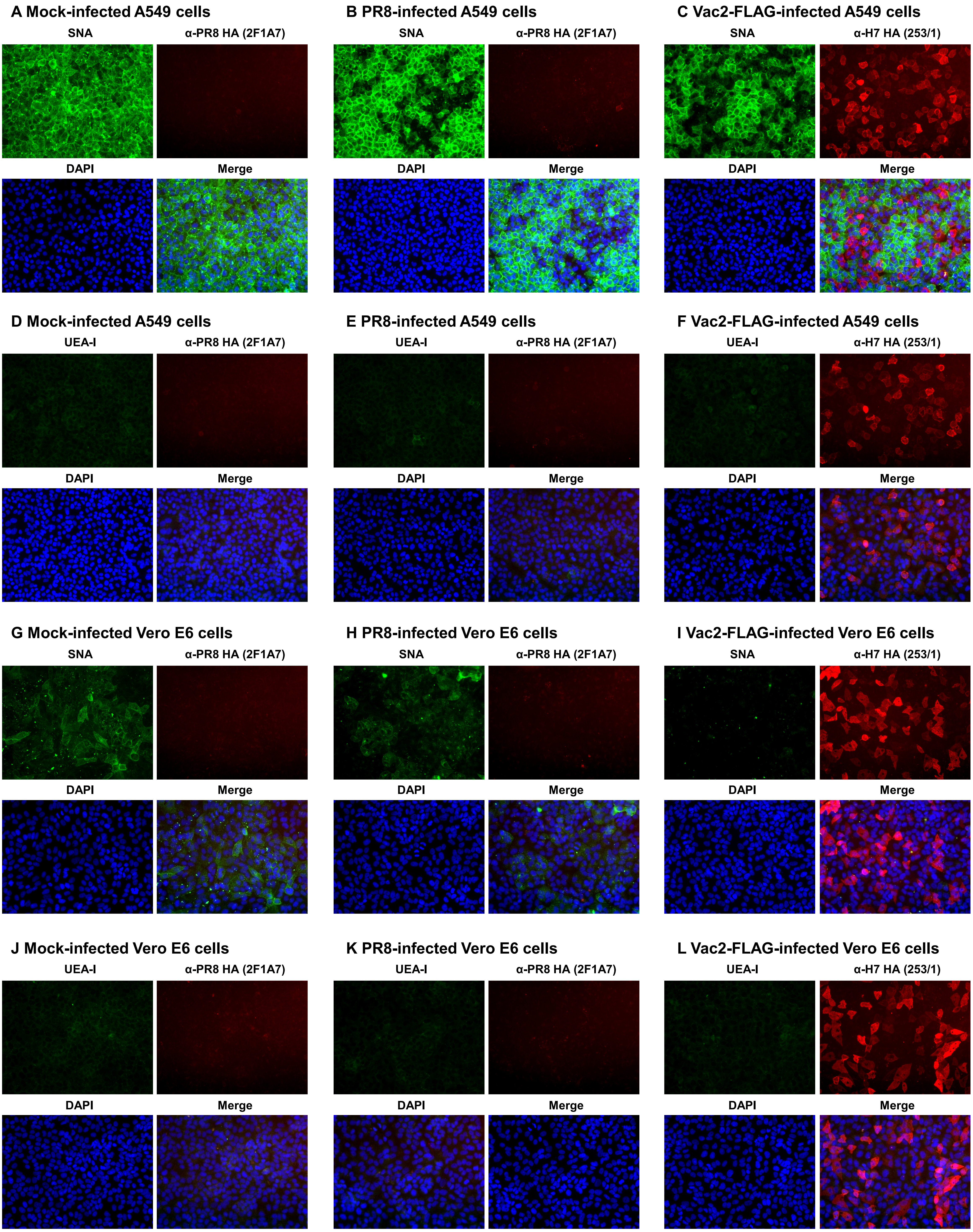

Fig S3

Kobayashi *et al*

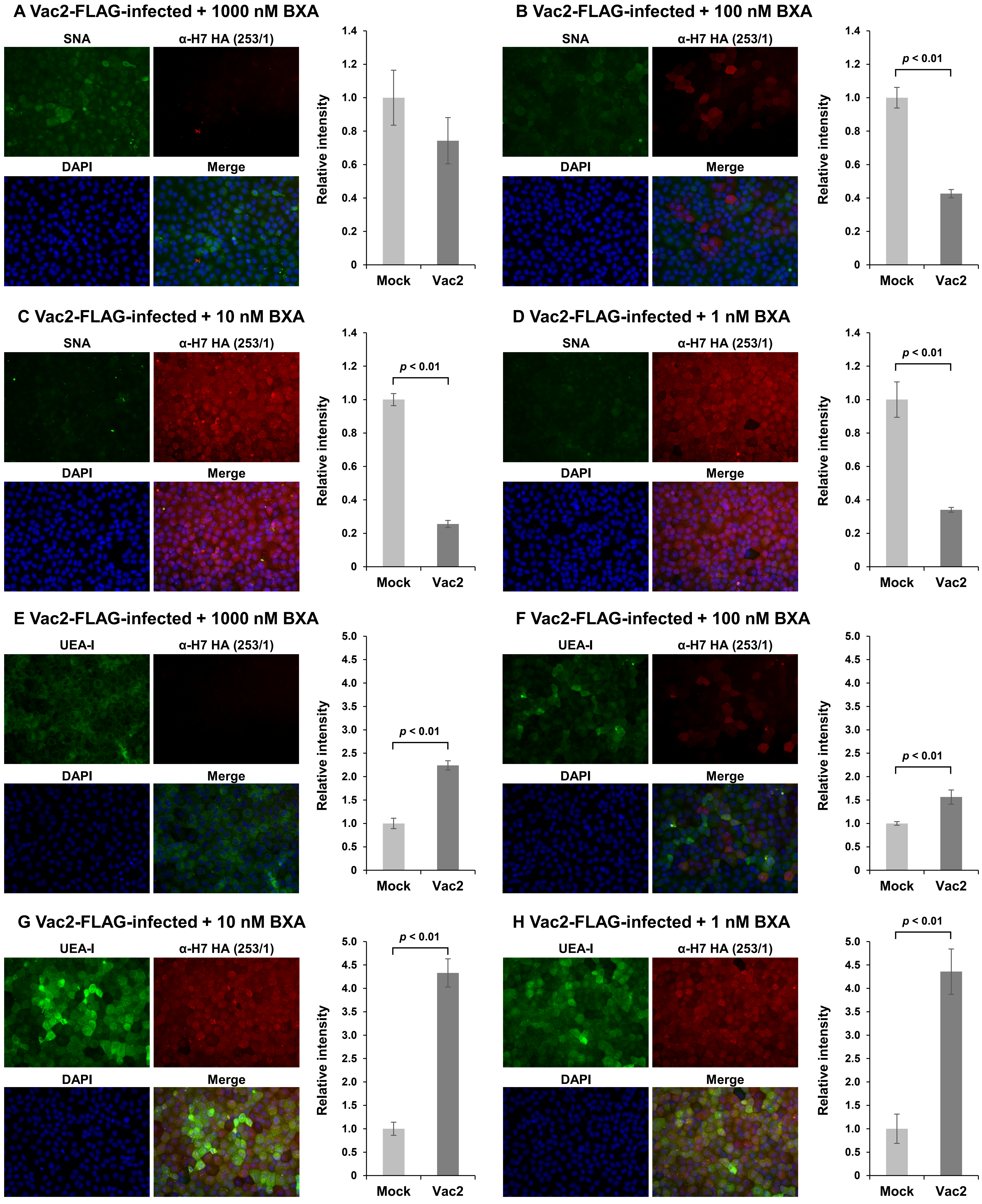

Fig S4

Kobayashi *et al*

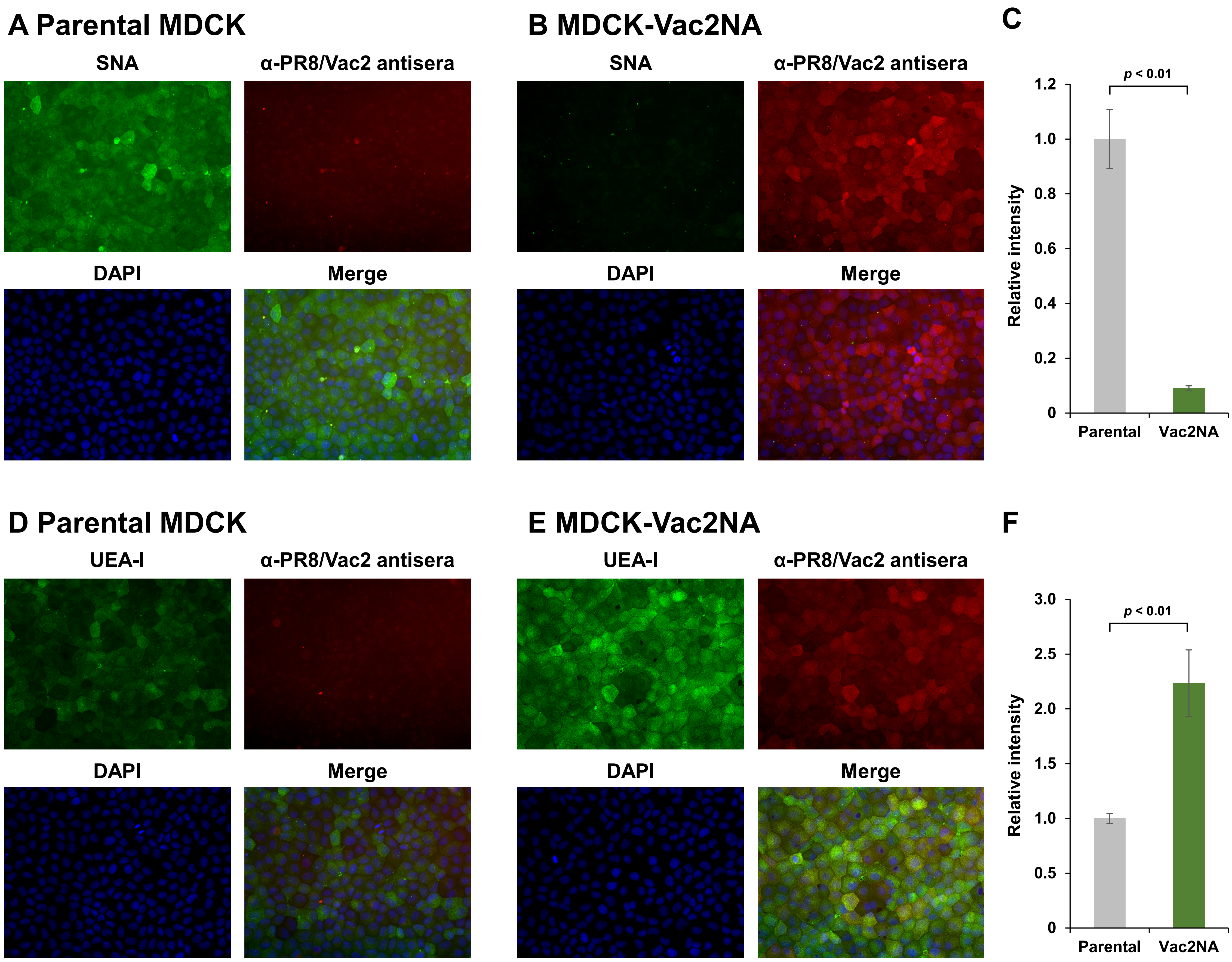

Fig S5

Kobayashi *et al*

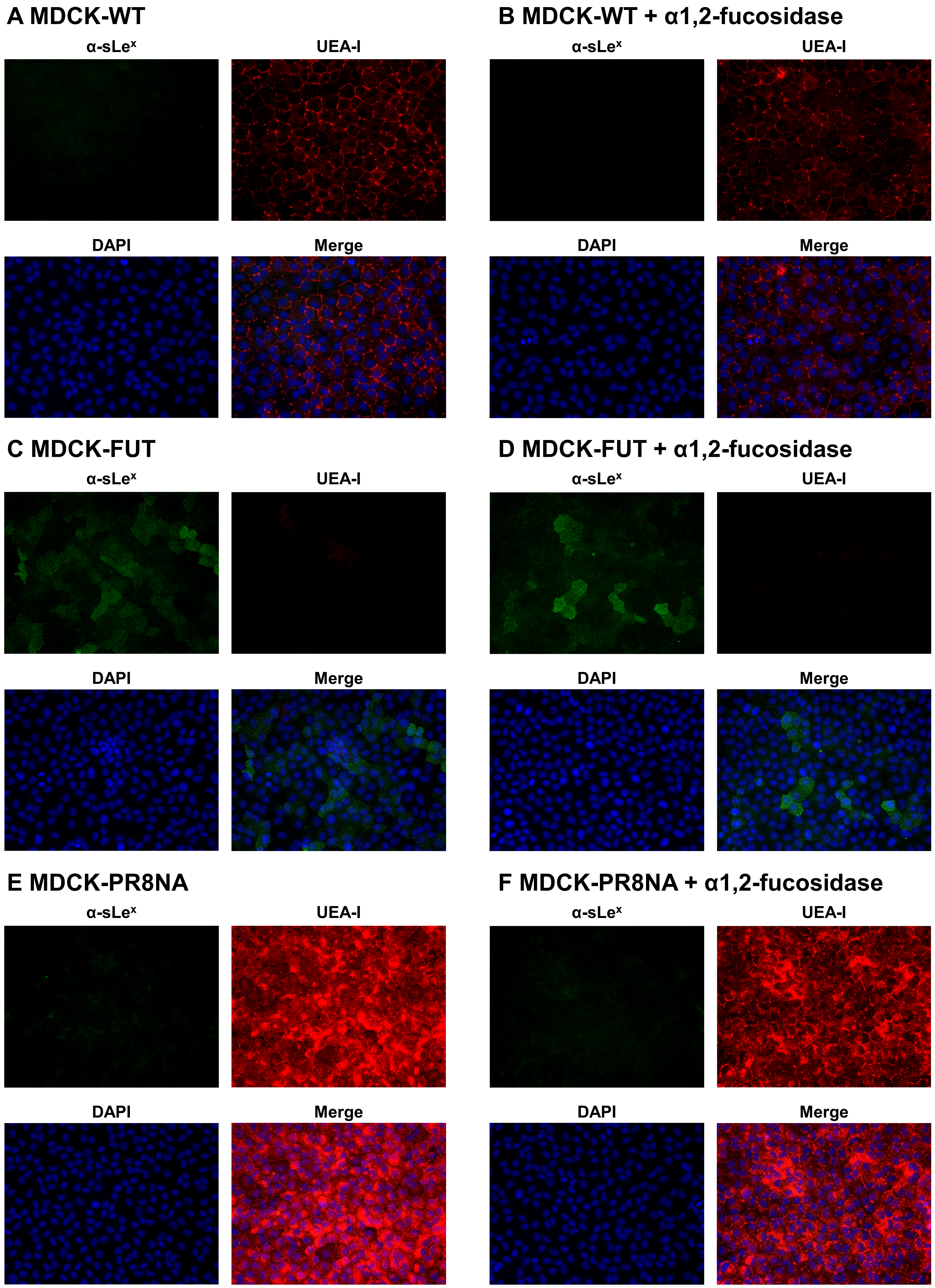

Fig S6

Kobayashi *et al*

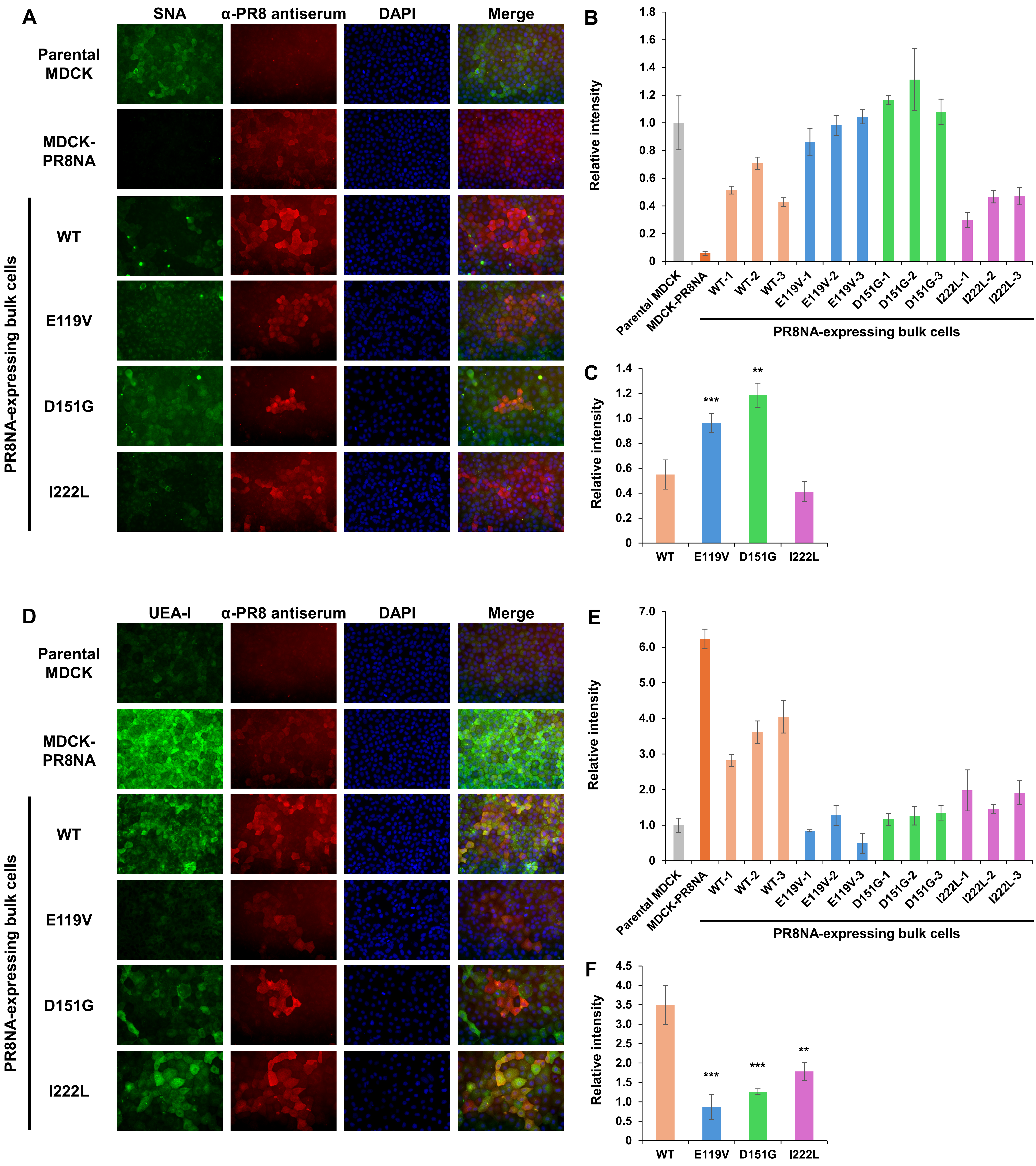

Fig S7

Kobayashi *et al*

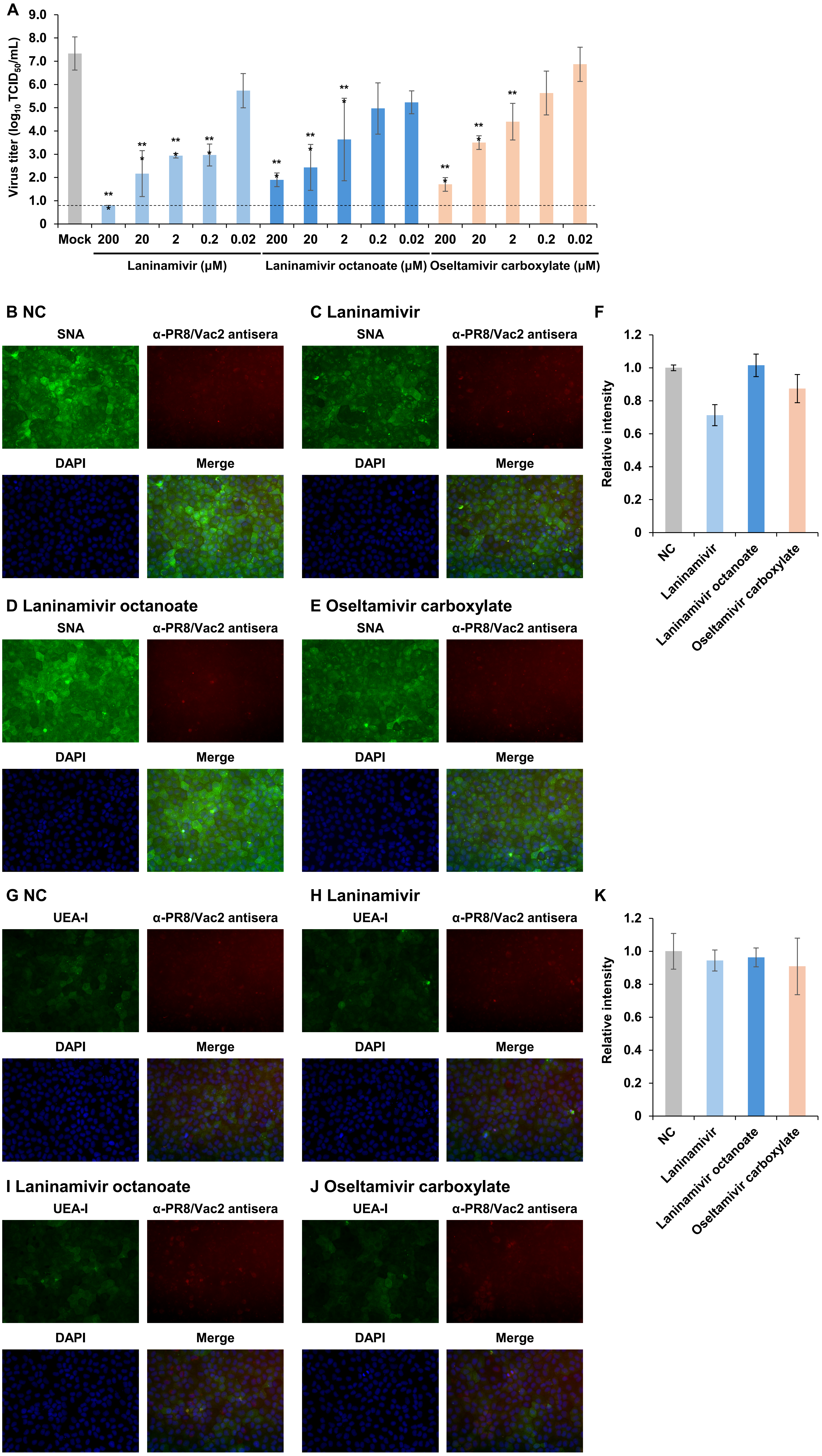

Fig S8 **Kobayashi *et al***

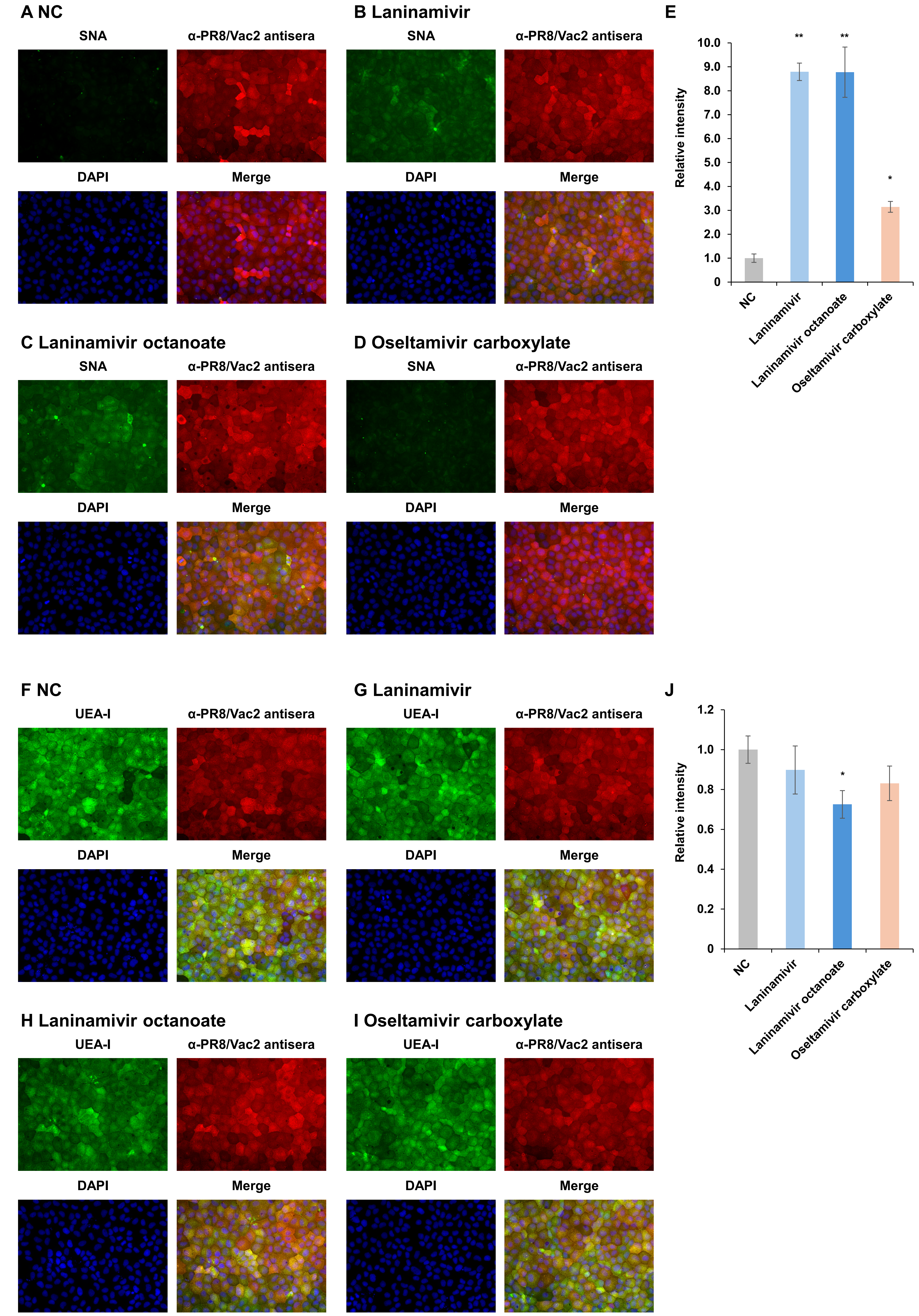

Fig S9

Kobayashi *et al*

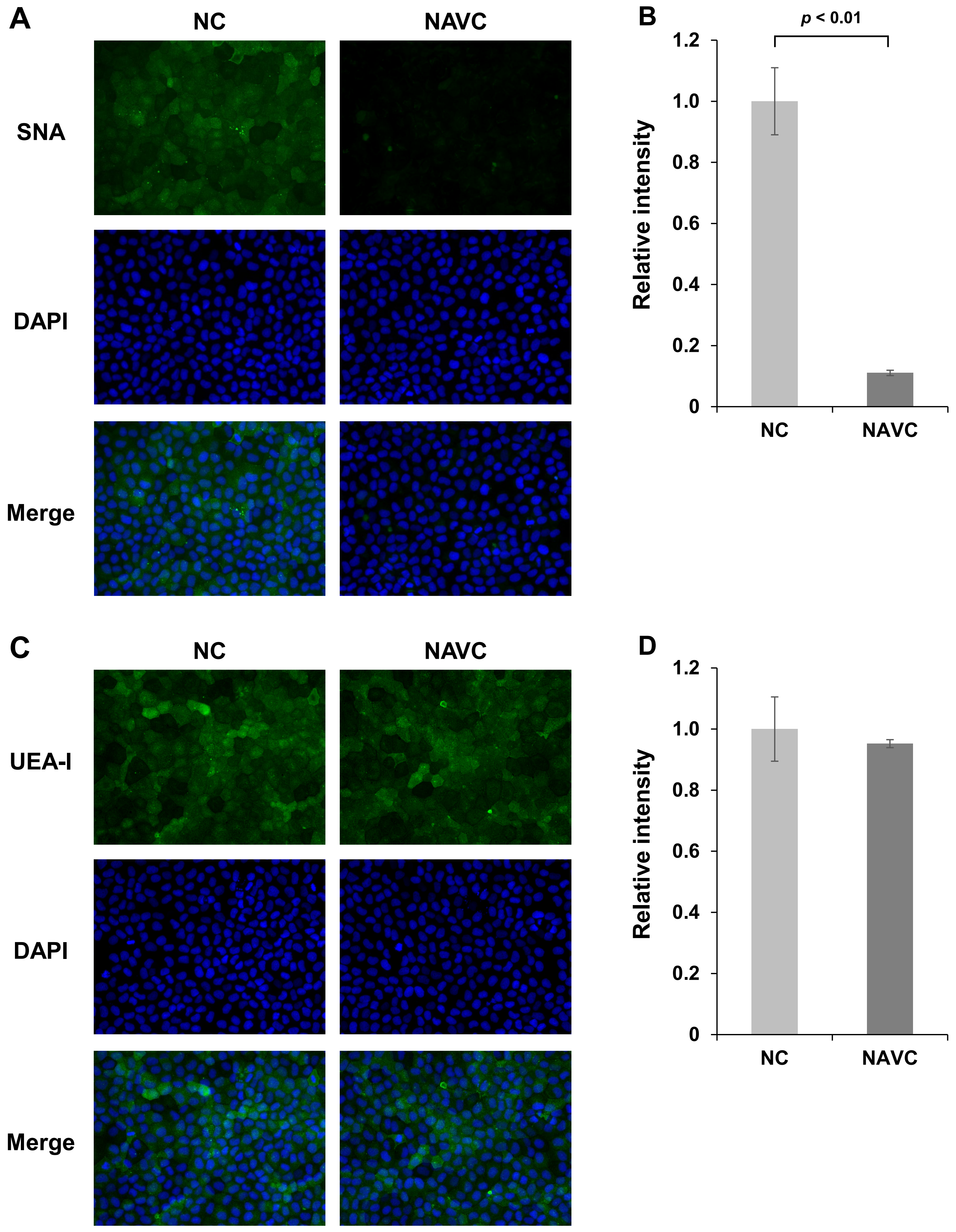

Fig S10

Kobayashi *et al*

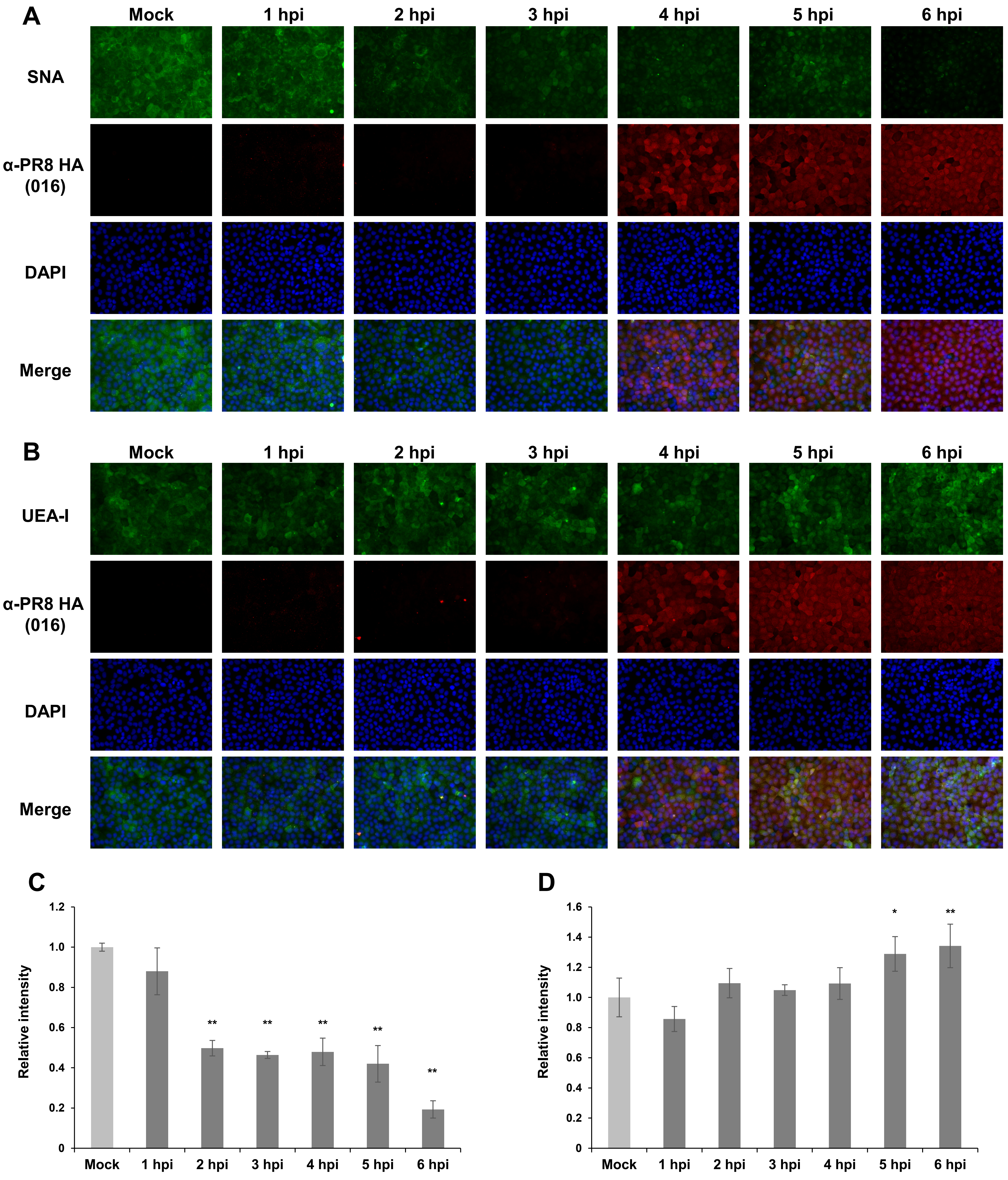

Fig S11

Kobayashi *et al*

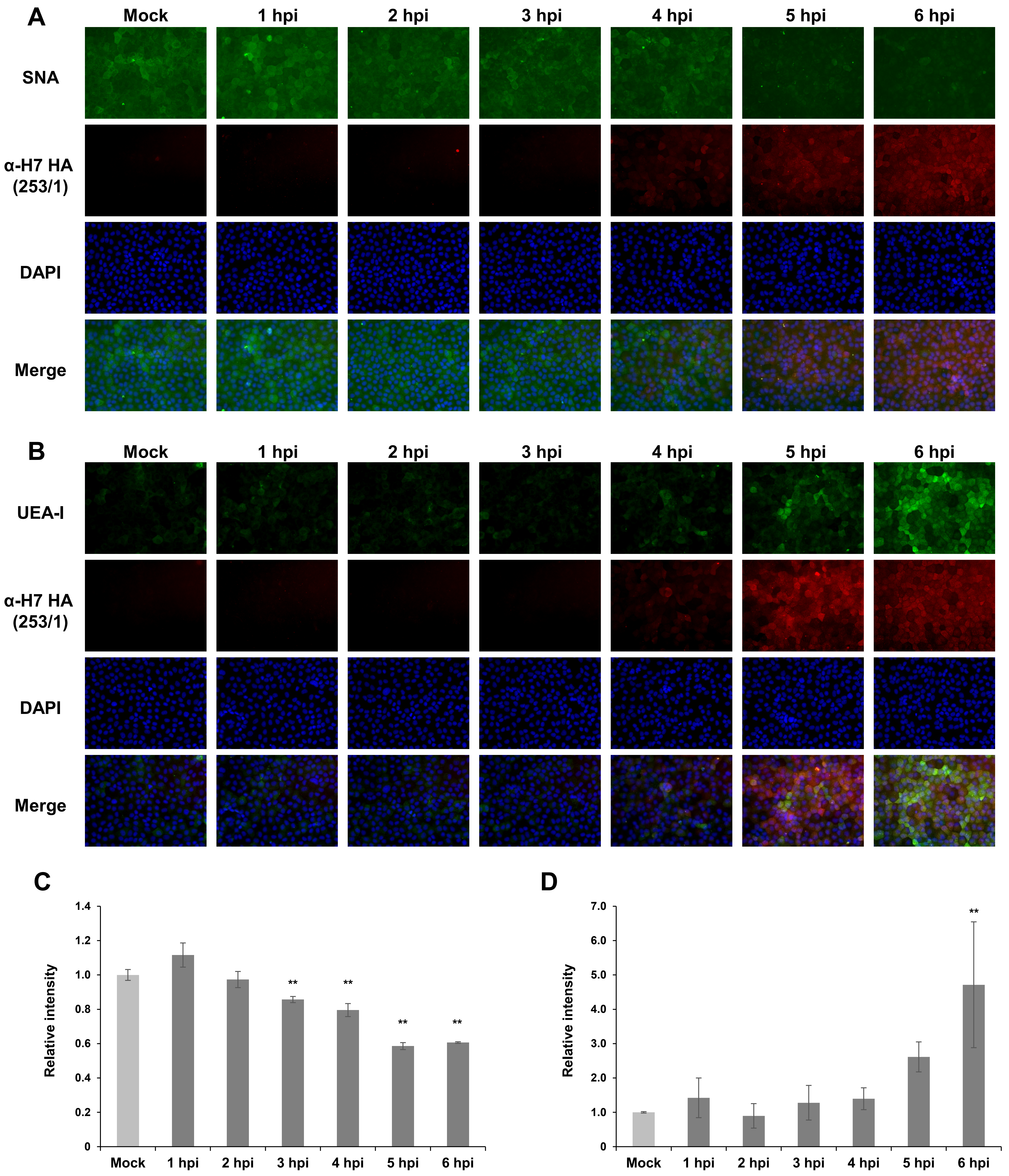

Fig S12

Kobayashi *et al*

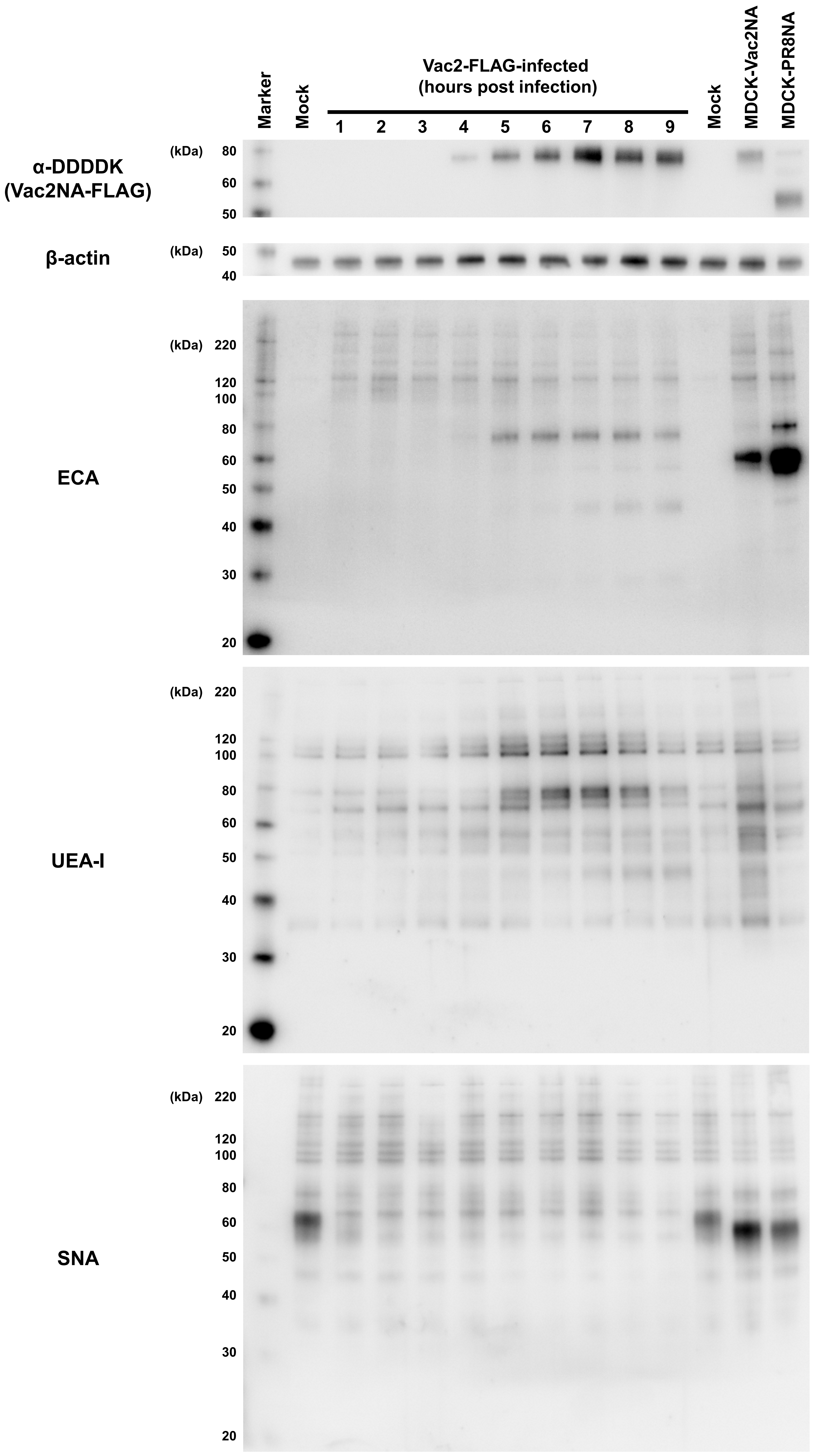

Fig S13 **Kobayashi *et al***
