# Supplementary material for: Neuraminidase of influenza A viruses induces global desialylation of host cells via its intracellular function
Source: Microbiol Spectr. 2026 Feb 18;14(4):e03328-25. doi: 10.1128/spectrum.03328-25 (PMC13055363; doi:10.1128/spectrum.03328-25)
Supplement: Supplemental material — Supplemental figure legends. [file spectrum.03328-25-s0002.docx]

**Supplementary materials**

**Fig S1.** **SNA staining of MDCK cells infected with various AIVs.** (A–J) Lectin and immunofluorescence staining of AIV (N1–9 subtypes) or mock-infected MDCK cells at 8 hpi. (K) Fluorescent intensities derived from SNA in an average of three fields of view were compared. The signal intensities of IAV-infected cells compared to that of mock-infected ones were statistically analyzed by one-way ANOVA with Dunnett’s post hoc test (*: *p* < 0.05, **: *p* < 0.01, ***: *p* < 0.001).

**Fig S2.** **UEA-I staining of MDCK cells infected with various AIVs.** (A–J) Lectin and immunofluorescence staining of AIV (N1–9 subtypes) or mock-infected MDCK cells at 8 hpi. (K) Fluorescent intensities derived from UEA-I in an average of three fields of view were compared. The signal intensities of IAV-infected cells compared to that of mock-infected ones were statistically analyzed by one-way ANOVA with Dunnett’s post hoc test (*: *p* < 0.05, **: *p* < 0.01, ***: *p* < 0.001).

**Fig S3.** **Lectin staining of A549 and Vero E6 cells infected with PR8 or Vac2 virus.** (A–F) Lectin and immunofluorescence staining of IAV or mock-infected A549 cells at 8 hpi. (G–L) Lectin and immunofluorescence staining of IAV or mock-infected Vero E6 cells at 8 hpi.

**Fig S4.** **Inhibitory effect of BXA on glycan alterations on Vac2-infected MDCK cells.** MDCK cells infected with Vac2 were cultured in the presence of 1–1000 nM of BXA for 8h and subjected to lectin and immunofluorescence staining. (A–D) The cells were stained with SNA (A–D; green) or UEA-I (E–H; green) and an anti-H7 HA monoclonal antibody (red), and counterstained with DAPI (blue). Fluorescent intensities derived from SNA or UEA-I in an average of three fields of view were compared between mock- and Vac2-infected cells. The statistical analysis was performed with Student’s t-test.

**Fig S5.** **Desialylation and α1-2 fucosylation in MDCK cells stably expressed NA protein of Vac2 virus.** (A, B) The wild type and Vac2 NA-expressing MDCK cells were stained with SNA (green) and immunostained with a mixture of PR8 and Vac2 chicken antisera (red) and counterstained with DAPI (blue). (C) Fluorescent intensities derived from SNA in an average of three fields of view were compared between mock- and Vac2-infected cells. The statistical analysis was performed with Student’s t-test. (D, E) The wild type and Vac2 NA-expressing MDCK cells were stained with UEA-I (green) and immunostained with a mixture of PR8 and Vac2 chicken antisera (red) and counterstained with DAPI (blue). (F) Fluorescent intensities derived from UEA-I in an average of three fields of view were compared between mock- and Vac2-infected cells. The statistical analysis was performed with Student’s t-test.

**Fig S6. Expression of NA protein induced α1-2 fucosylation without upregulation of sialyl Lewis X antigen.** Wild-type MDCK cells were co-stained with an anti-sialyl Lewis X monoclonal antibody (green) and UEA-I (red) with (B) or without (A) pretreatment with α1-2 fucosidase. MDCK cells overexpressing a chicken fucosyltransferase (cFUT3/5/6) gene (C, D) and NA-expressing MDCK cells (E, F) were also costained with the anti-sialyl Lewis X antibody (green) and UEA-I (red) with or without pretreatment with α1-2 fucosidase and also counterstained with DAPI (blue).

**Fig S7. Glycan alterations in MDCK cells expressing catalytic-dead NA. MDCK cells were transfected with** wild-type (WT) or catalytic-dead mutants (E119V, D151G, and I222L) of PR8 NA, and G418-resistant populations were analyzed. (A) Representative images of SNA staining. MDCK and MDCK-PR8NA represent parental and cloned PR8 NA-expressing MDCK cells, respectively. SNA signal intensities of G418-resistant bulk cells expressing PR8 NA mutants (WT, E119V, D151G, I222L) were compared. Cells expressing WT and I222L at higher levels exhibited lower SNA signals. (B) Fluorescence intensities of SNA were quantified from the average of three fields of view per sample across three biologically independent replicates. (C) Mean SNA signal intensities from three biological replicates were compared between WT and each mutant. Statistical analysis was performed using one-way ANOVA followed by Dunnett’s post hoc test (*: *p* < 0.05, **: *p* < 0.01, ***: *p* < 0.001). (D) Representative images of UEA-I staining. MDCK and MDCK-PR8NA represent parental and cloned PR8 NA-expressing MDCK cells, respectively. UEA-I signal intensities of G418-resistant bulk cells expressing PR8 NA mutants (WT, E119V, D151G, I222L) were compared. Cells expressing WT and I222L at higher levels exhibited lower SNA signals. (E) Fluorescence intensities of UEA-I were quantified from the average of three fields of view per sample across three biologically independent replicates. (F) Mean UEA-I signal intensities from three biological replicates were compared between WT and each mutant. Statistical analysis was performed using one-way ANOVA followed by Dunnett’s post hoc test (*: *p* < 0.05, **: *p* < 0.01, ***: *p* < 0.001).

**Fig S8. Treatment with NA inhibitors did not induce glycan alterations in MDCK cells.** (A) Antiviral effect of NA inhibitors (laninamivir, laninamivir octanoate, oseltamivir carboxylate) was assessed by culturing the PR8-infected cells in the presence of several concentrations of the inhibitors. The dotted line indicates the threshold for virus titration (<0.8 log_10_ TCID_50_/mL). To compare the viral titers between mock- and NA inhibitor-treated groups, statistical analysis was performed using the one-way ANOVA with Dunnett’s post hoc test (*: *p* < 0.05, **: *p* < 0.01, ***: *p* < 0.001). Virus titers below the detection limit were considered as 0.8 log_10_ TCID_50_/mL for statistical analysis. (B–E) MDCK cells treated with NA inhibitor were stained with SNA. (G–J) MDCK cells treated with NA inhibitor were stained with UEA-I. Fluorescent intensities derived from SNA (F) and UEA-I (K) in an average of three fields of view were compared to non-treated control (NC). The statistical analysis was performed using the one-way ANOVA with Dunnett’s post hoc test (*: *p* < 0.05, **: *p* < 0.01, ***: *p* < 0.001).

**Fig S9.** **Glycan alterations induced by treatment with NA inhibitors in MDCK-Vac2NA cells.** (A–D) MDCK-Vac2NA cells treated with NA inhibitor were stained with SNA. (F–I) MDCK-Vac2NA cells treated with NA inhibitor were stained with UEA-I. Fluorescent intensities derived from SNA (E) and UEA-I (J) in an average of three fields of view were compared to non-treated control (NC). The statistical analysis was performed using the one-way ANOVA with Dunnett’s post hoc test (*: *p* < 0.05, **: *p* < 0.01, ***: *p* < 0.001).

**Fig S10.** **Bacterial neuraminidase treatment in MDCK cells diminished sialic acids while it did not upregulate representation of α1-2 fucose.** (A, C) MDCK cells were treated with recombinant neuraminidase derived from *Vibrio* *cholerae* (NAVC). The cells were stained with SNA and UEA-I (green) and counterstained with DAPI (blue). (B, D) The signal intensities of each lectin in the neuraminidase-treated cells were compared with those in the non-treated cells (NC). The statistical analysis was performed with Student’s t-test.

**Fig S11.** **Glycan profile changes observed on the surface of MDCK cells during PR8 infection.** MDCK cells infected with PR8 at an MOI of 1 were fixed in 4% paraformaldehyde for 3–6 hpi. The cells were then stained with either SNA (A) or UEA-I (B) for glycan profiling (green), and HA protein expression was detected using an anti-PR8 HA monoclonal antibody (red) and also counterstained with DAPI (blue). (C, D) The signal intensities of the lectins were statistically analyzed by one-way ANOVA with Dunnett’s post hoc test (*: *p* < 0.05, **: *p* < 0.01, ***: *p* < 0.001).

**Fig S12.** **Glycan profile changes observed on the surface of MDCK cells during Vac2 infection.** MDCK cells infected with Vac2-FLAG at an MOI of 1 were fixed in 4% paraformaldehyde for 3–6 hpi. The cells were stained with either SNA (A) or UEA-I (B) for glycan profiling (green), and the expression of NA proteins was detected using an anti-DDDDK-tag monoclonal antibody (red). and also counterstained with DAPI (blue). (C, D) The signal intensities of the lectins were statistically analyzed by one-way ANOVA with Dunnett’s post hoc test (*: *p* < 0.05, **: *p* < 0.01, ***: *p* < 0.001).

**Fig S13.** **Time-dependent NA protein expression and glycan alterations in MDCK cells infected with Vac2-FLAG.** Whole lysates of Vac2-FLAG-infected MDCK cells were assessed by western blotting and lectin blotting. NA protein and β-actin expressed in MDCK cells were detected with anti-DDDDK and β-actin antibodies, respectively.

**Table S1. List of 45 lectins used in lectin microarray analyses.**

**Table S2. Primer list used for RT-qPCR of glycosyltransferase genes.**
